# Intestinal DMBT1 Expression Is Modulated by Crohn’s Disease-Associated *IL23R* Variants and by a *DMBT1* Variant Which Influences Binding of the Transcription Factors CREB1 and ATF-2

**DOI:** 10.1371/journal.pone.0077773

**Published:** 2013-11-05

**Authors:** Julia Diegelmann, Darina Czamara, Emmanuelle Le Bras, Eva Zimmermann, Torsten Olszak, Andrea Bedynek, Burkhard Göke, Andre Franke, Jürgen Glas, Stephan Brand

**Affiliations:** 1 Department of Medicine II - Grosshadern, Ludwig-Maximilians-University (LMU), Munich, Germany; 2 Department of Preventive Dentistry and Periodontology, Ludwig-Maximilians-University (LMU), Munich, Germany; 3 Max-Planck-Institute for Psychiatry, Munich, Germany; 4 Munich Cluster for Systems Neurology (SyNergy), Munich, Germany; 5 Division of Gastroenterology, Hepatology and Endoscopy, Brigham and Women’s Hospital, Harvard Medical School, Boston, Massachusetts, United States of America; 6 Department of Clinical Chemistry, Ludwig-Maximilians-University (LMU), Munich, Germany; 7 Institute of Clinical Molecular Biology, Christian-Albrechts-University, Kiel, Germany; 8 Department of Human Genetics, Rheinisch-Westfälische Technische Hochschule (RWTH), Aachen, Germany; Dr. Margarete Fischer-Bosch and University of Tübingen, Germany

## Abstract

**Objectives:**

DMBT is an antibacterial pattern recognition and scavenger receptor. In this study, we analyzed the role of *DMBT1* single nucleotide polymorphisms (SNPs) regarding inflammatory bowel disease (IBD) susceptibility and examined their functional impact on transcription factor binding and downstream gene expression.

**Methods:**

Seven SNPs in the *DMBT1* gene region were analyzed in 2073 individuals including 818 Crohn’s disease (CD) patients and 972 healthy controls in two independent case-control panels. Comprehensive epistasis analyses for the known CD susceptibility genes *NOD2*, *IL23R* and *IL27* were performed. The influence of *IL23R* variants on DMBT1 expression was analyzed. Functional analysis included siRNA transfection, quantitative PCR, western blot, electrophoretic mobility shift and luciferase assays.

**Results:**

IL-22 induces DMBT1 protein expression in intestinal epithelial cells dependent on STAT3, ATF-2 and CREB1. IL-22 expression-modulating, CD risk-associated *IL23R* variants influence DMBT1 expression in CD patients and DMBT1 levels are increased in the inflamed intestinal mucosa of CD patients. Several *DMBT1* SNPs were associated with CD susceptibility. SNP rs2981804 was most strongly associated with CD in the combined panel (p = 3.0×10^−7^, OR 1.42; 95% CI 1.24–1.63). All haplotype groups tested showed highly significant associations with CD (including omnibus *P*-values as low as 6.1×10^−18^). The most strongly CD risk-associated, non-coding *DMBT1* SNP rs2981804 modifies the DNA binding sites for the transcription factors CREB1 and ATF-2 and the respective genomic region comprising rs2981804 is able to act as a transcriptional regulator *in vitro*. Intestinal DMBT1 expression is decreased in CD patients carrying the rs2981804 CD risk allele.

**Conclusion:**

We identified novel associations of *DMBT1* variants with CD susceptibility and discovered a novel functional role of rs2981804 in regulating DMBT1 expression. Our data suggest an important role of DMBT1 in CD pathogenesis.

## Introduction

The pathogenesis of the inflammatory bowel diseases (IBD) Crohn’s disease (CD) and ulcerative colitis (UC) is not completely understood. Current models of disease pathogenesis suggest that IBD is caused by a complex interplay of environmental factors such as the microbiota of the host, the host immune system and genetic predisposition. The first IBD susceptibility gene *NOD2*/*CARD15* (which is particularly associated with small intestinal disease), was identified in 2001 and encodes an intracellular bacterial pattern recognition receptor (PRR) [1], [2]. Since then, emerging evidence suggests that particularly autophagy genes such as *ATG16L1* [3], [4] and *IRGM* [5], [6] as well as genes involved in the proinflammatory Th17 cell pathway such as *IL23R, IL12B, JAK2, STAT3, CCR6,* and *IL2/IL21* [7], [8], [9], [10] are associated with CD.

DMBT1, a glycoprotein of the scavenger receptor cystein-rich (SRCR) family, is a protein participating in antibacterial host defense on mucosal surfaces such as the intestine or the oral mucosa. It acts as a PRR that recognizes leucine-rich repeats of bacterial proteins [11]. It is known that DMBT1 inhibits NF-κB activation induced by the bacterial products lipopolysaccharide (LPS) and muramyldipeptide (MDP) [12], aggregates group A *Streptococci* by binding pili [13], and inhibits *Salmonella enterica* cytoinvasion [12], resulting in reduced bacterial adhesion to human epithelial cells [13], [14], [15], [16]. Interestingly, DMBT1 is a target gene of NOD2 [12] and also a target of TLR4 for which we and others demonstrated associations with IBD susceptibility [17], [18].

Studies with experimental dextran sulfate sodium (DSS) colitis models in DMBT1 knockout (KO) mice had heterogeneous outcomes. While in one study, no difference between KO and wild-type (WT) mice was observed [19], another study reported enhanced susceptibility to DSS colitis in KO mice [20] whereas a third study observed differences only at low doses of DSS [16]. However, to what extent these findings can be directly transferred to human IBD, remains to be determined. The contradictory results may reflect a general problem in using mouse models for human intestinal inflammation as it has been demonstrated recently that some mouse models of inflammation poorly mimic the more complex human conditions [21].

In humans, it has been demonstrated that DMBT expression is upregulated by proinflammatory stimuli such as TNF-α or LPS [12] and correlates with disease activity in IBD patients [20], [22]. Recently, a copy number polymorphism in *DMBT1*, resulting in a substantially decreased number of scavenger receptor cysteine-rich (SRCR) domains, was identified as a risk factor for CD [20].

In this study, we aimed to further clarify the role of DMBT1 in intestinal epithelial cells (IEC) and in the pathogenesis of IBD. We investigated the IL-22-mediated transcriptional regulation of DMBT1 in the IEC lines HT-29 and DLD-1 and analyzed the influence of IL-22 expression-modulating, CD-associated *IL23R* variants on DMBT1 expression in biopsies from CD patients. Moreover, we analyzed seven *DMBT1* gene variants and their haplotypes, including four so far not analyzed single nucleotide polymorphisms (SNPs) in a large panel of 2073 Caucasian individuals regarding their association with IBD risk and disease phenotype. We tested for gene-gene interaction (epistasis) of *DMBT1* variants with known CD susceptibility variants in *NOD2*, *IL23R* and *IL27* since previous studies demonstrated that these genes may also be involved in the modulation of DMBT1 expression [12], [23], [24]. In addition, we analyzed the functional impact of variants in *DMBT1* on transcription factor binding to the respective DNA and the downstream gene expression and identified a CD-associated *DMBT1* variant that is linked to the colonic DMBT1 gene expression in CD patients.

## Patients and Methods

### Ethics Statement

The study was approved by the Ethics committee of the Medical Faculty of the Ludwig-Maximilians-University Munich. Written, informed consent was obtained from all patients prior to the study. Study protocols were based on the ethical principles for medical research involving human subjects of the Helsinki Declaration (http://www.wma.net/e/policy/b3.htm).

### Reagents and Antibodies

Human recombinant IL-22 was obtained from R&D Systems (Wiesbaden, Germany). Antibodies against CREB1 and ATF-2 were from Santa Cruz Biotechnology (Heidelberg, Germany) and p84 antibody was from Abcam (Cambridge, UK). The secondary anti-rabbit antibody was from GE Healthcare (Freiburg, Germany).

### RNA Isolation, Reverse Transcription and Quantitative PCR

Total RNA from IEC was isolated using the RNeasy Mini Kit from Qiagen (Hilden, Germany) and 500 ng were reverse transcribed using the Transcriptor First Strand cDNA Synthesis Kit from Roche (Mannheim, Germany). Total RNA from intestinal biopsies was isolated using a Branson sonifier to disrupt the tissue followed by RNA extraction with Trizol reagent and chloroform. Quantitative real-time PCR was performed as previously described [23] using a LightCycler 480 instrument (Roche Diagnostics, Mannheim, Germany) and SYBR green detection format. Gene expression was normalized to the β-actin expression in the respective samples. The following primers were used for quantification: CREB1 forward 5′-CACAGATTGCCACATTAGCC-3′, CREB1 reverse 5′-TGAACTGTTTGGACTTGTGGAG-3′, ATF-2 forward 5′-GTCATGGTAGCGGATTGGTT-3′, ATF-2 reverse 5′-CTTCTCCGACGACCACTTGT-3′, DMBT1 forward 5′-TGCTCTGTCTGCCAAATCAC-3′, DMBT1 reverse 5′-GTCATTGTCTGCCTGCTTGA-3′, β-actin forward 5′-CCTCGCCTTTGCCGATCCGC-3′, β-actin reverse 5′-CCACCATCACGCCCTGGTGC-3′.

### Western Blot

Western Blot analysis was performed according to standard procedures [25] with nuclear extracts isolated from IEC lines as described [23]. Briefly, 20 µg of nuclear extract or 50 µg of total protein were separated on an 8–16% gradient polyacrylamide gel and were transferred to a PVDF membrane. Membranes were blocked with 5% milk in TBS-T and incubated with the primary antibody overnight at 4°C. Following incubation with the secondary HRP-coupled antibody, luminescent detection was performed with the ECL system (Pierce) and a CCD camera (Peqlab, Erlangen, Germany).

### siRNA Transfection

DLD-1 cells were reverse transfected in 24 well plates (for RNA isolation) or 10 cm plates (for nuclear extract isolation) with siRNA (Life Technologies, Darmstadt, Germany) using Lipofectamine RNAiMAX (Life Technologies) following the manufacturer’s guidelines. Total mRNA and nuclear protein was isolated and specific knockdown was assessed by quantitative PCR and Western Blot, respectively.

### *In silico* Analysis of Transcription Factor Binding Sites

Genomic sequences including SNPs rs2981745 and rs2981804 were analyzed for potential transcription factor binding sites of human transcription factors with the TFSEARCH program (http://www.cbrc.jp/research/db/TFSEARCH.html) which is based on the TRANSFAC database [26]. The threshold score for binding site prediction was set to 75.0 (score = 100.0*(‘weighted sum’−min)/(max−min); max. score = 100). For each SNP, both alleles including the flanking sequences 10 bp upstream and downstream were compared.

### Nuclear Extracts and Electrophoretic Mobility Shift Assay (EMSA)

Isolation of nuclear extracts from IEC lines HT-29, DLD-1, HCT116 and SW480 was performed according to standard procedures [27] with minor modifications [23]. EMSA analysis was performed essentially as described [23]. Briefly, 100 fmol of biotinylated, double-stranded probes were incubated with 5 µg of nuclear extract from cell lines as indicated in the presence of 1 µg of poly d(I)-d(C) for 30 min. Where indicated, 1 µg of anti-CREB1 or anti-ATF-2 antibody or a non-specific negative control antibody was added. For competition experiments, 50-fold molar excess of unlabelled probe was included in the reaction before adding the labelled probe. Samples were separated on a 6% polyacrylamide gel (2.5 hours at 100 V for the DMBT1 probe, 1.5 hours for the CREB1 probe) and were transferred to a positively charged nylon membrane. Detection of biotinylated probes was performed with streptavidin-HRP using the LightShift Chemiluminescent EMSA kit from Pierce (Thermo Fisher Scientific, Bonn, Germany). The sequences of the two different DMBT1 probes used were 5′-CCTGCTAACGTAACCAAATTGCTA-3′ and 5′-CCTGCTAACGTAGCCAAATTGCTA-3′. The sense orientation for each probe is given and the polymorphic nucleotide comprising *DMBT1* SNP rs2981804 is underlined. The sequence for the CREB1 probe was 5′-AGAGATTGCCTGACGTCAGAGAGCTAG-3′ and the sequence for the ATF-2 probe was 5′-CTTAGTTACGTAATAATTGT-3′ (consensus sequence underlined). The unspecific competitor probe had the sequence 5′-GATCCTTCTGGGCCGTCCTAGATC-3′.

### Plasmid Cloning, Transient Transfection and Luciferase Assay

The genomic region harboring SNP rs2981804 was amplified by PCR from HT-29 cells (genotype AA) or DLD1 cells (genotype GG). The resulting fragments were cloned either into the luciferase reporter plasmid pGL4.23 (Promega, Mannheim, Germany) which contains a minimal promoter (minP) in front of the luciferase gene resulting in low basal expression or into the pGL4.13 vector with a strong SV40 promoter/enhancer with high basal expression. For both vector backbones, three constructs were cloned: 1) DMBT1 insert upstream, i.e. 5′ of the promoter into the multiple cloning site, 2) DMBT1 insert 2 kb downstream, i.e. 3′ of the promoter, and 3) DMBT1 insert 3′ and 5′ of the promoter. All constructs were verified by sequencing.

DLD1 or HT-29 cells were transfected in 96-well plates with 100 or 200 ng of plasmid, respectively, using Lipofectamine LTX (Life Technologies), together with 10 or 20 ng of Renilla luciferase plasmid. After 24 hours, luciferase activity was detected using the Dual-Glo Luciferase Assay System (Promega). Relative light units (RLU) were normalized to the levels of Renilla luciferase. A detailed description of all experimental cloning procedures and the primers used can be found in the methods S1, figures S1 and S2 and table S1.

### Study Population

The study population (n = 2073) consisted of 818 Crohn’s disease (CD) patients and 972 healthy, unrelated controls [(623 CD patients and 762 controls in the discovery panel (recruited from the IBD center of the University Hospital Munich-Grosshadern) and 195 CD patients and 210 controls in the replication panel (from the LMU Munich Innenstadt Campus)]. In addition, we analyzed 283 UC patients recruited from the IBD center of the University Hospital Munich-Grosshadern. All patients were of Caucasian origin.

Phenotypic data were collected blind to the results of the genotypic data and consisted of demographic and clinical parameters (behaviour and anatomic location of IBD, disease-related complications, surgical or immunosuppressive therapy) which were recorded by two senior gastroenterologists using patient charts analysis and a detailed questionnaire including an interview at time of enrolment. The diagnosis of CD and UC was based on established guidelines based on endoscopic, radiological, and histopathological parameters. The phenotypic classification of CD patients was based on the Montreal classification [28], including age at diagnosis (A), location (L), and behaviour (B) of disease. In patients with UC, anatomic location was also assessed in accordance to the Montreal classification based on the criteria ulcerative proctitis (E1), left-sided UC (distal UC; E2), and extensive UC (pancolitis; E3). Patients with indeterminate colitis were excluded from the study. The demographic characteristics of the study population are presented in table 1.

**Table 1:** Demographic and phenotypic characteristics of the IBD study population.

	Crohn’s disease	Ulcerative colitis	Controls
	n = 818	n = 283	n = 972
**Gender**			
Male (%)	46.0	53.0	63.8
Female (%)	54.0	47.0	36.2
**Age** (yrs)			
Mean ± SD	40.7±13.3	43.8±14.8	46.0±10.3
Range	15–81	17–88	19–68
**Body mass index**			
Mean ± SD	23.0±4.2	23.9±4.5	
Range	13–41	15–54	
**Age at diagnosis** (yrs)			
Mean ± SD	27.9±12.0	31.3±13.7	
Range	6–78	4–81	
**Disease duration** (yrs)			
Mean ± SD	13.1±8.8	11.9±8.43	
Range	0–46	1–50	
**Positive family history of IBD** (%)	16.6	17.4	
**CD: Age at onset** (n = 731)			
≤16 years (A1)	188 (25.7%)		
17–40 years (A2)	461 (63.1%)		
>40 years (A3)	82 (11.2%)		
**CD: Disease location** (n = 777)			
Terminal ileum (L1)	113 (14.5%)		
Colon (L2)	99 (12.7%)		
Ileocolon (L3)	555 (71.4%)		
Upper GI (L4)	10 (1.3%)		
**CD: Behavior** (n = 755)			
Non-stricturing, non-penetrating (B1)	185 (24.5%)		
Stricturing (B2)	210 (27.8%)		
Penetrating (B3)	360 (47.7%)		
**UC: Disease location (UC)** (n = 261)			
Proctitis (E1)		24 (9.2%)	
Left-sides colitis (E2)		97 (37.2%)	
Pancolitis (E3)		140 (53.6%)	

For each criterion according to the Montreal classification, the number of patients for which data were available, is given.

### Sampling of Intestinal Biopsies from Patients with Crohn’s Disease

In a subgroup of 27 CD patients, 75 intestinal biopsies from inflamed and non-inflamed intestinal tissue were collected during routine endoscopy. Written informed consent was obtained from all patients prior to biopsy sampling. The study was approved by the Ethics Committee of the Medical Faculty of the Ludwig-Maximilians-University Munich. Detailed patient characteristics are summarized in table 2.

**Table 2:** Characteristics of the CD patients from which intestinal biopsies were collected.

Patient ID	Number of biopsies	Anatomic location of biopsy sampling*	*DMBT1* rs2981804 genotype	Location Montreal L	Behavior Montreal B	NOD2 mutation‡
1	2	1x sigma (−), 1x colon (−)	AA	1	3	1007fs +/+
2	2	1x colon (−), 1x colon (−)	AA	3	3	G908R +/−
3	1	1x sigma (−)	AA	3	1	−
4	5	3x colon (−), 1x sigma (−), 1x rectum (−)	GA	3	2	−
5	2	1x sigma (−), 1x sigma (+)	GA	3	2	−
6	5	1x colon (−), 4x colon (+)	GA	3	2	R702W +/−, G908R +/−
7	2	1x rectum (−), 1x colon (+)	GA	3	3	1007fs +/+
8	1	1x colon (−)	GA	4	2	−
9	2	1x colon (−), 1x colon (+)	GA	3	2	−
10	2	1x colon (−), 1x cecum (+)	GA	3	1	−
11	2	2x colon (−)	GA	3	3	R702W+/−, G908R+/−
12	2	1x colon (−), 1x ileocecal valve (+)	GA	3	1	−
13	2	1x colon (−), 1x colon (+)	GA	2	2	−
14	2	2x colon (−)	GA	3	2	R702W+/−
15	1	1x colon (−)	GG	3	2	−
16	1	1x cecum (−)	GG	3	3	1007fs+/+
17	3	1x colon (−), 2x colon (+)	GA	3	3	1007fs+/−
18	4	2x colon (−), 2x colon (+)	AA	3	3	−
19	4	2x cecum (−), 2x cecum (+)	AA	3	3	−
20	4	2x cecum (−), 2x terminal ileum (+)	AA	3	1	−
21	3	2x colon (−), 1x colon (+)	AA	3	2	1007fs+/+
22	4	2x cecum (−), 2x terminal ileum/ileocecal valve (+)	AA	3	2	−
23	4	2x colon (−), 2x colon (+)	AA	3	3	G908R+/−
24	4	2x colon (−), 2x terminal ileum (+)	AA	3	3	G908R+/−
25	4	2x cecum (−), 2x terminal ileum (+)	AA	3	2	−
26	3	2x colon (−), 1x colon (+)	GA	3	1	−
27	4	2x cecum (−), 2x cecum (+)	GA	3	3	−
**total**	**n = 75**	**n = 45 (**−**)**	**AA: 11 (41%)**	**L1∶1 (4%)**	**B1∶5 (19%)**	**R702W: 3 (11%)**
		**n = 30 (+)**	**GA: 14 (52%)**	**L2∶1 (4%)**	**B2∶11 (41%)**	**G908R: 3 (11%)**
			**GG: 2 (7%)**	**L3∶24 (89%)**	**B3∶11 (41%)**	**1007fs: 7 (26%)**
				**L4∶1 (4%)**		**NOD2 positive: 11 (41%)**

* (−) = non-inflamed tissue; (+) = inflamed tissue;

‡ given are the three main *NOD2* mutations R702W, G908R, 1007fs; − = no mutation, +/− = heterozygous mutation, +/+ homozygous mutation.

### DNA Extraction and Genotyping

For genotyping, genomic DNA was isolated from peripheral blood leukocytes from all study participants using the DNA blood mini kit (Qiagen, Hilden, Germany) following the manufacturer’s instructions. The study participants were genotyped for seven SNPs in the *DMBT1* gene region.

**Figure 1:**
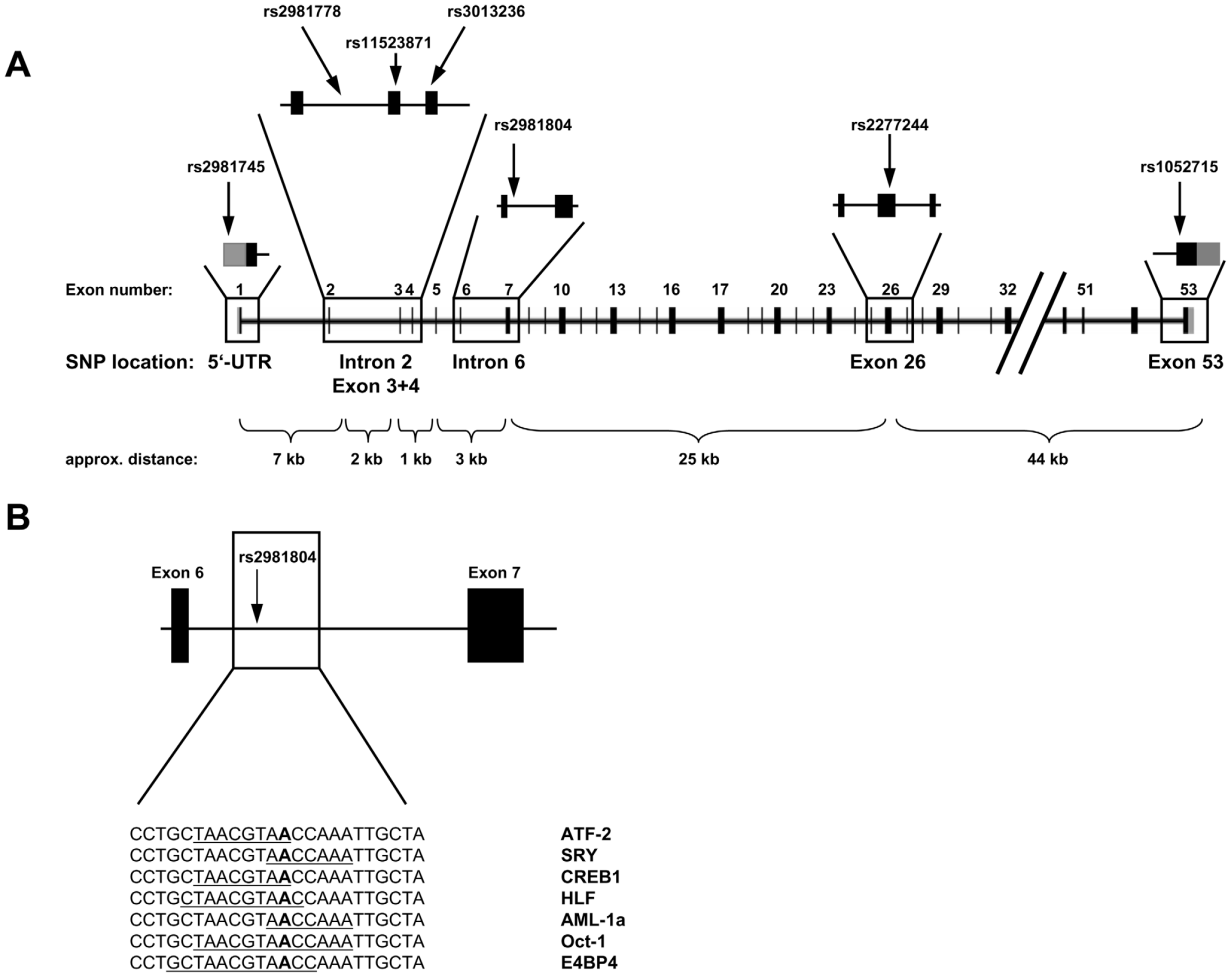
Overview of the *DMBT1* gene structure, SNP location and predicted transcription factor binding sites. (A) Exon-intron structure of the *DMBT1* gene and location of the analyzed *DMBT1* SNPs. grey = untranslated region. (B) Potential binding sites of transcription factors in the genomic region comprising SNP rs2981804. The binding sites on the genomic DNA are underlined; the polymorphic nucleotide is depicted in bold underlined.

The *DMBT1* SNPs rs2981745, rs3013236 (corresponds to p.Leu54Ser) and rs1052715 (p.Pro1707Pro) were investigated in the study of Renner and co-workers [20]. Additionally, the tagging SNPs rs2981778, rs11523871 (p.Pro42Thr), rs2981804 and rs2277244 (p.His585Tyr) were selected from the data of the International HapMap project covering the *DMBT1* gene plus 10 kb flanking the centromeric and telomeric end of the gene, respectively, and using a setting of r^2^ of 0.8 (Fig. 1A). Genotyping was performed by PCR and melting curve analyses using a pair of fluorescence resonance energy transfer (FRET) probes in a LightCycler480 instrument. Detailed genotyping methodology and the primers/probes used for genotyping are summarized in the methods S1 and in tables S2 and S3. In addition, detailed haplotype and subphenotype analyses were performed. Gene-gene interactions (epistasis) of *DMBT1* variants with variants in *NOD2*, *IL23R* and *IL27* were analyzed. Those genotype data were available from previous studies [8], [29], [30], [31], [32], [33]. IL-22 serum protein levels were analyzed in previous study [34].

### Statistical Analysis

Single-marker allelic tests were performed with Pearson’s χ^2^ test. Student’s t-test was applied for quantitative variables. All tests were two-tailed and *P-*values <0.05 were considered nominally significant. Correction for multiple testing was performed using Bonferroni’s method. We tested 7 *DMBT* SNPs for association with two phenotypes (CD and UC status) resulting in a corrected *P*-value of 0.0036 (0.05/(2*7)). Empirical *P*-values were derived using 10,000,000 point-wise permutations. Odds ratios (OR) were calculated for the risk allele at each SNP. Data were evaluated by using the SPSS 13.0 software (SPSS Inc., Chicago, IL, U.S.A.) and R-2.4.1. (http://cran.r-project.org). Permutation-based *P*-values, interaction *P*-values as well as haplotype and LD analysis were conducted using PLINK v 1.07 (http://pngu.mgh.harvard.edu/~purcell/plink/). We ran a sliding window approach, varying the window size from 2 to 7 included markers and using the option “hap-logistic”. Haplotype omnibus *P*-values are based on jointly testing all *H* haplotype effects at the specific position resulting in a *H-1* degrees of freedom test. For haplotype specific tests, individuals presenting with the specific haplotype are compared to all other individuals.

## Results

### The Th17 Cytokine IL-22 Up-regulates STAT3-dependent DMBT1 Expression in Intestinal Epithelial Cells

**Figure 2:**
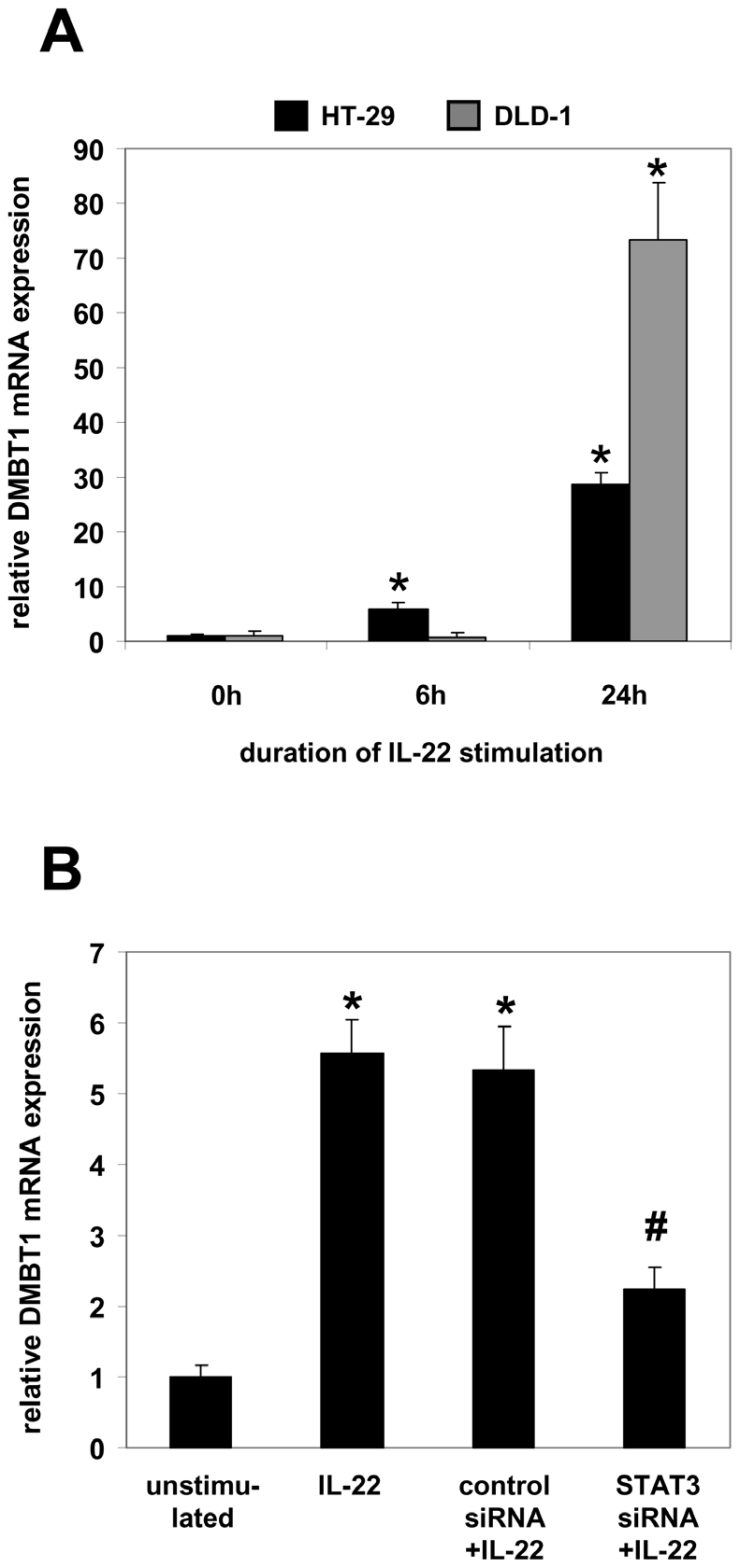
The Th17 cytokine IL-22 induces DMBT1 expression in intestinal epithelial cells dependent on STAT3. (A) DMBT1 expression is induced by IL-22 in intestinal epithelial cells as determined by quantitative PCR in HT-29 and DLD-1 cells stimulated with 100 ng/ml IL-22 for 6 and 24 hours, respectively. Data are from five independent experiments. DMBT1 expression in unstimulated cells was arbitrarily set to 1.0 and expression in IL-22-stimulated cells was calculated accordingly. *p<0.01 vs. unstimulated cells. (B) Silencing of STAT3 expression by siRNA transfection reduces IL-22-induced DMBT1 expression in DLD-1 cells. *p<0.01 vs. unstimulated; #p<0.05 vs. control+IL-22.

We and others have shown that IL-22 is an inducer of antimicrobial peptides like defensins in IEC and other tissues [35], [36], [37]. DMBT1 is an antibacterial scavenger receptor (9) and we recently demonstrated that the STAT3-activating cytokine IL-27 induces DMBT1 expression in IEC [23]. Given that IL-22 is a known STAT3 activator [35], we hypothesized that IL-22 might induce DMBT1 expression in IEC. To test this hypothesis, we stimulated HT-29 and DLD-1 cells with 100 ng/ml IL-22 for 6 and 24 hours, respectively. Quantitative PCR revealed a significant up-regulation of DMBT1 expression in both cell lines (Fig. 2A). To determine the signaling pathways involved, we transfected DLD-1 cells with siRNA against STAT3 or an unspecific control siRNA prior to IL-22 stimulation. The IL-22-induced DMBT1 expression was significantly reduced in cells with knocked-down STAT3 expression (Fig. 2B).

### Crohn’s Disease-associated *IL23R* Variants, which Modulate IL-22 Expression, Influence Intestinal DMBT1 Expression in Patients with Crohn’s Disease

**Figure 3:**
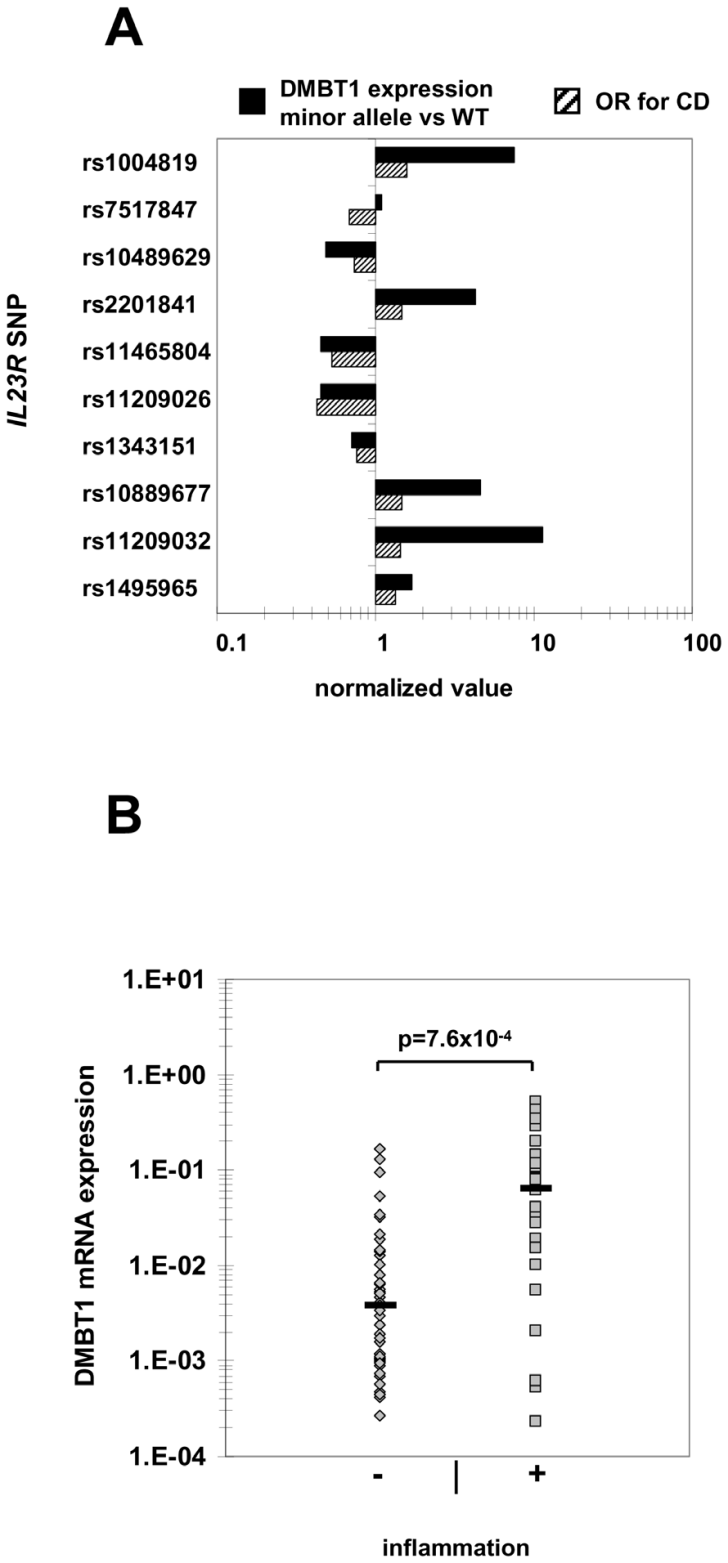
*IL23R* CD risk-increasing variants are associated with higher DMBT1 expression in minor allele carriers and intestinal inflammation increases DMBT expression levels in CD patients. (A) Intestinal DMBT1 expression and *IL23R* genotypes were determined in 75 biopsies from 27 CD patients. DMBT1 expression is presented as a quotient derived from dividing expression in minor allele carriers of the respective *IL23R* SNP by the expression in WT carriers. ORs for the respective SNPs were available from a previous study [8]. (B) Intestinal DMBT1 expression is significantly higher in inflamed colonic biopsies (n = 30) compared to not inflamed regions (n = 45) from 27 CD patients. Expression was normalized to β-actin in the respective samples. Each dot represents one biopsy.

We have recently demonstrated that CD-associated variants in *IL23R* modulate IL-22 expression. Higher IL-22 serum levels were found in carriers of CD risk-associated *IL23R* SNPs and lower IL-22 serum levels in carriers of CD-protective *IL23R* SNPs [34] (summarized in table 3). Having shown that IL-22 induces DMBT1 expression, we further analyzed these *IL23R* variants regarding their association with DMBT1 expression. DMBT1 mRNA expression was determined by quantitative PCR in a total of n = 75 inflamed and not inflamed intestinal biopsies collected from 27 CD patients (for patient characteristics, see table 2). DMBT1 expression in carriers of the minor allele (homozygous and heterozygous) of the respective *IL23R* SNPs was divided by the expression in homozygous carriers of the wild-type (WT) allele (table 3, Fig. 3A). The odds ratios (ORs) for the analyzed *IL23R* SNPs were available from a previous study [8]. In 5 out of 10 *IL23R* SNPs, there were significant differences (p<0.05) in DMBT1 expression between carriers of the minor allele and WT carriers (table 3). Similar to our previous results obtained for IL-22 [34], there was a high correlation of OR regarding CD susceptibility and DMBT expression ratio minor allele vs. WT (r _(OR/DMBT) = _0.766) and between IL-22 and DMBT1 ratios minor allele vs WT (r_(DMBT/IL-22) = _0.754). For all SNPs except rs7517847, patients with risk-increasing *IL23R* variants had higher DMBT1 expression when carrying the minor allele (OR>1 and relative DMBT1 expression minor allele vs WT>1), while in patients with IBD risk-decreasing *IL23R* variants, DMBT1 expression was lower in minor allele carriers compared to WT carriers (OR<1 and relative expression minor vs. WT<1; table 3, Fig. 3A). The correlation of OR and DMBT1 expression was independent of the inflammation status of the analyzed biopsies (figure S3).

**Table 3:** *IL23R* gene variants modulate intestinal DMBT1 expression.

(1)	(2)	(3)	(4)	(5)	(6)	(7)
*IL23R* SNP	genotype (no of biopsies)	MedianDMBT1expression	*P*-valueminorallele vs WT	DMBT1 expressionfor *IL23R* minorallele vs WT	IL-22 expressionfor *IL23R* minorallele vs WT^#^	OR regarding CD suscep-tibility^‡^
rs1004819	CC (n = 13)	0.00210	**0.002**	7.53	1.05	1.56
	CT (n = 48) TT (n = 14)	0.01585				
rs7517847	TT (n = 35)	0.01029	0.504	1.09	1.05	0.68
	TG (n = 38) GG (n = 2)	0.01120				
rs10489629	AA (n = 33)	0.01905	0.183	0.48	0.96	0.73
	AG (n = 41) GG (n = 2)	0.00916				
rs2201841	TT (n = 21)	0.00412	**0.044**	4.29	1.06	1.46
	TC (n = 37) CC (n = 17)	0.01767				
rs11465804	TT (n = 74)	0.01120	n.d.	0.45	0.81	0.53
	TG (n = 1) GG (n = 0)	0.00505				
rs11209026	GG (n = 74)	0.01120	n.d.	0.45	0.72	0.43
	GA (n = 1) AA (n = 0)	0.00505				
rs1343151	CC (n = 46)	0.01440	0.401	0.71	0.87	0.76
	CT (n = 27) TT (n = 2)	0.01029				
rs10889677	GG (n = 21)	0.00412	**0.039**	4.60	1.07	1.47
	GA (n = 36) AA (n = 17)	0.01895				
rs11209032	GG (n = 18)	0.00167	**8**×**10** ^−**4**^	11.32	1.26	1.43
	GA (n = 40) AA (n = 17)	0.01895				
rs1495965	AA (n = 5)	0.00661	**0.033**	1.69	1.10	1.33
	AG (n = 50) GG (n = 20)	0.01120				

The median DMBT1 mRNA expression was analyzed in a total of 75 biopsies from 27 CD patients for each *IL23R* variant. *P* values in column (4) are given for the comparison of the DMBT1 expression of carriers of the *IL23R* minor allele (homozygous and heterozygous) compared to DMBT1 levels in *IL23R* wild-type carriers. Column (5) summarizes the data from column (3) and represents the fold increase or decrease in DMBT1 expression in carriers of the *IL23R* minor allele (homozygous and heterozygous) compared to DMBT1 levels in *IL23R* wild-type carriers for the respective *IL23R* SNP. The IL-22 serum levels of column 6 summarizes the results of a previous study [34] while the odds ratios of column 7 represent the results of a previous detailed genotype analysis [8], in which all patients analyzed here participated. The correlation coefficient between the DMBT1 quotient of column (5) and the ORs of column (7) is r = 0.766.

### Intestinal DMBT1 Expression is Increased in Active Crohn’s Disease

Having demonstrated that DMBT1 is up-regulated by IL-22 and IL-27 [23] and given the increased expression of IL-22 and IL-27 in active IBD as shown by us and others [34], [35], [38], we next compared DMBT1 mRNA expression levels in all inflamed (n = 30 biopsies) and not inflamed (n = 45 biopsies) intestinal biopsies from the above described 27 CD patients. DMBT1 expression was significantly higher (16.6-fold) in inflamed colonic biopsies compared to not inflamed regions (Fig. 3B, p = 7.6×10^−4^).

### Associations of *DMBT1* Gene Variants and Haplotypes with Crohn’s Disease in the German Population

Having shown that DMBT1 expression is associated with disease activity in CD patients, we next aimed to determine whether SNPs in the *DMBT1* gene region might influence IBD susceptibility. We genotyped 7 *DMBT1* SNPs (rs2981745, rs2981778, rs11523871 [p.Pro42Thr], rs3013236 [p.Leu54Ser], rs2981804, rs2277244 [p.His585Tyr], rs1052715 [p.Pro1707Pro]) in a cohort of 818 CD patients and 972 controls (composed of two separate cohorts: discovery panel: n = 623 cases/762 controls, replication panel: n = 195 cases/210 controls). Allele frequencies of all SNPs were in Hardy-Weinberg equilibrium (P-value>0.05 after Bonferroni correction for multiple testing; table S4). For all case-control panels, we demonstrated significant disease associations with certain *DMBT1* SNPs (table 4, table S5). In the combined CD sample panel (table 4), the most strongly CD-associated SNP was rs2981804 (p = 3.0×10^−7^, odds ratio (OR) 1.42, 95% confidence interval (CI) [1.24–1.63]), followed by SNP rs2981745 (p = 7.7×10^−3^, OR 1.21, 95% CI [1.05–1.39]; table 4). In addition, the SNPs rs2981778, rs11523871 and rs3013236 (in linkage disequilibrium (LD) with rs2981745, table S6) were weakly associated with CD. Moreover, we genotyped a panel of 283 UC patients (table S7). Both most strongly CD-associated *DMBT1* SNPs were also associated with UC but rs2981745 displayed the stronger association (rs2981745: p = 2.5×10^−4^, OR 1.50, 95% CI [1.24–1.82], rs2981804: p = 2.5×10^−3^, OR 1.31 [1.08–1.58]; table S7).

**Table 4:** Association results of *DMBT1* gene variants with CD in the combined discovery and replication panels.

		Crohn’s disease: Combined panel n = 818			Controls n = 972
SNP	Risk allele	RAF	empirical *P*-value	OR [95% CI]	RAF
rs2981745	T	0.372	**7.7**×**10** ^−**3**^	1.21 [1.05–1.39]	0.329
rs2981778	G	0.702	**0.034**	1.16 [1.01–1.35]	0.669
rs11523871 = p.Pro42Thr	A	0.703	**0.038**	1.16 [1.01–1.33]	0.671
rs3013236 = p.Leu54Ser	T	0.708	**0.020**	1.19 [1.03–1.37]	0.670
rs2981804	A	0.563	***3.0*** *×* ***10*** ^−***7***^	1.42 [1.24–1.63]	0.475
rs2277244 = p.His585Tyr	C	0.976	0.086	1.41 [0.92–2.13]	0.966
rs1052715 = p.Pro1707Pro	A	0.575	0.11	1.12 [0.98–1.28]	0.547

Risk allele frequencies (RAF), allelic test empirical *P*-values (1 degree of freedom), and odds ratios (OR, shown for the riskallele) with 95% confidence intervals (CI) are depicted for both the CD and UC case-control panels. *P*-values <0.05 are highlighted in **bold** and *P*-values robust to multiple testing (P<0.0036) are highlighted in ***bold italic***. P-values are based on 10,000,000 permutations.

To elucidate if the two most strongly IBD-associated SNPs represent independent disease association, we conditioned association analysis on the strongest signal, i.e. rs2981804 for CD and rs2981745 for UC. Even after conditioning, effects for rs2981745 in CD (p = 1.0×10^−5^) and rs2981804 in UC (p = 4.5×10^−4^) remained significant. Moreover, there was only weak LD between both SNPs (r^2^ = 0.24, 0.18 and 0.17 for CD, UC, and controls, respectively). All allele frequencies, *P-*values and ORs are shown in tables 4 and tables S5–S7. Next, potential phenotypic consequences of the two *DMBT1* variants rs2981745 and rs2981804 were investigated using the Montreal classification of CD and UC [28]. However, all statistical significant phenotypic associations were rather weak and were not robust to multiple testing (table S8–S10).

In order to investigate for potential disease associations with certain *DMBT1* haplotypes, we performed a detailed haplotype analysis considering all possible haplotypes with a frequency of at least 1% in the whole sample (table S11 and S12). In CD patients, all haplotype groups analyzed were significantly associated with the disease after correction for multiple testing including corrected omnibus *P-*values of <10^−10^ in 60% of all analyzed haplotype groups (table S11). The strongest association with CD was found for a haplotype consisting of all seven analyzed SNPs (omnibus *P-*value: 6.14×10^−18^, table 5 and table S11). This association was mainly attributable to two haplotypes: CGATGCA and CGATACA, which differ only in the rs2981804 allele (underlined). The rare CGATGCA haplotype was significantly more common in controls (haplotype frequency (HF) 0.13) than in CD patients (HF 0.02; p = 4.04×10^−13^; OR 0.25, 95% CI [0.17–0.36]). The more abundant haplotype CGATACA was significantly more prevalent in CD patients (HF: 0.34; p = 1.53×10^−8^, OR 1.54, 95% CI [1.22–1.79] than in controls (HF 0.17).

**Table 5:** *DMBT1* haplotypes are associated with CD.

Haplotype	Crohn’s disease			Controls
	HF	*P-*value	OR [95% CI]	HF
rs2981745–rs2981778–rs11523871–rs3013236–rs2981804–rs2277244–rs1052715		***6.14***×***10*** ^−***18***^		
CGATGTG	0.01	9.88×10^−2^	0.62 [0.33–1.14]	0.02
CGATACG	0.13	**7.33**×**10** ^−**3**^	0.77 [0.64–0.93]	0.23
CGATGCG	0.09	**2.81**×**10** ^−**2**^	0.78 [0.63–0.97]	0.10
TACCGCA	0.15	1.17×10^−1^	0.85 [0.70–1.04]	0.20
TACCACA	<0.01	6.85×10^−2^	<0.01 [<0.01–1.50]	0.03
TACCGCG	0.11	6.57×10^−1^	0.95 [0.74–1.21]	0.06
CGATGCA	0.02	***4.04***×***10*** ^−***13***^	0.25 [0.17–0.36]	0.13
CGATACA	0.34	***1.53***×***10*** ^−***8***^	1.54 [1.33–1.79]	0.17
TGATACG	0.03	***2.92***×***10*** ^−***5***^	29.40 [6.03–143.3]	<0.01

Haplotype frequencies (HF), *P*-values (including omnibus *P*-values), and odds ratios (OR) with 95% confidence intervals (CI) are given for the strongest haplotype-disease association. Only the most significantly associated haplotypes are shown. The complete haplotype data can be found in the supplementary material. *P-*values for individual haplotypes are presented for all haplotypes with a frequency of at least 1% in the whole sample and with an omnibus haplotype *P-*value <0.05. Significant *P-*values (<0.05) are highlighted in **bold** and significant *P*-values robust to multiple testing (*P*<2.5×10^−3^ *for omnibus P*-values, *P*<4.8×10^−4^ for detailed haplotype *P-*values) are highlighted in ***bold italic***.

### Analysis for Epistasis between *DMBT1* and *NOD2*, *IL23R* and *IL27* Gene Variants Regarding IBD Susceptibility

We then aimed to uncover potential epistasis of the *DMBT1* variants rs2981745 and rs2981804 with other replicated IBD susceptibility gene variants. We focused on *NOD2*, *IL23R* and *IL27*, given that DMBT1 is a target gene of NOD2 [12] and IL-23R [24]. Moreover, we recently demonstrated that *IL23R* modulates the expression of the Th17 cytokine IL-22 [34]. IL-22 is an epithelial barrier-protective cytokine [35] that is a transcriptional activator of DMBT1 expression as we demonstrated above. As IL-27 is a Th17 cell inhibiting cytokine [39], we also included variants in the *IL27* gene region for which we and others recently demonstrated an association with IBD susceptibility [32], [40]. Moreover, we have recently shown that DMBT1 is also a transcriptional target of IL-27 [23]. Data on *NOD2*, *IL23R* and *IL27* gene variants in our study population were available from previous studies [8], [32], [33]. No evidence for epistasis between *DMBT1* variants and SNPs in *NOD2* and the *IL27* gene region was found (table S13 and S14). For the *IL23R* SNP rs1004819, the most strongly IBD-associated *IL23R* SNP in our cohort [8], there was significant epistasis with the *DMBT1* SNPs rs2981745 (p = 0.042) and rs2981804 (p = 0.031) in UC but not CD patients (table S15). However, the identified statistical interaction was not robust to multiple testing.

### Allelic Variants of *DMBT1* SNP rs2981804 Alter the DNA Binding of the Transcription Factors CREB1 and ATF-2

As both most strongly IBD-associated *DMBT1* SNPs rs2981745 and rs2981804 are located in non-coding regions of *DMBT1* (rs2981745∶5′-untranslated region (UTR); rs2981804∶6th intron, Fig. 1A), we hypothesized that these SNPs might be located within DNA binding sequences of nuclear proteins. We therefore screened the respective genomic regions including the SNP and 10 bp upstream and downstream with the online tool TFsearch [26]. For rs2981745, no significant differences in DNA binding scores above the defined threshold were obtained for the two alleles (data not shown). For rs2981804, the program predicted differential binding of several transcription factors (table 6, Fig. 1B).

**Table 6:** Overview of potential transcription factor binding sites in the genomic region harboring the *DMBT* SNP rs2981804.

Factor	Consensus sequence#	Orientation(+ strand)	position relativeto SNP (5′ to 3′)	Binding scorerisk allele (A)	Binding score protective allele (G)
CRE-BP1 (ATF-2)	TTACGTA**A**	forward	−7 to 0	89.3	76.0
SRY	A**A**ACWAM	forward	−1 to+5	85.5	65.5
CREB1	TGACGTM**A**	forward	−7 to 0	84.2	77.7
HLF	R**T**TACRYAAT	reverse	+1 to−8	83.1	69.9
AML-1a	TGCGG**T**	reverse	+5 to 0	82.7	66.4
Oct-1	NNNRTA**A**TNANNN	forward	−7 to+5	79.3	70.0
E4BP4	NRTTAYGTA**A**YN	forward	−9 to+2	77.5	66.2

# Nucleotides in the consensus sequence that are identical to the genomic region surrounding SNP rs2981804 are underlined. The polymorphic nucleotide is depicted in bold. The orientation of the binding site is given based on the sequence of the + strand.

Binding score threshold for the risk allele was set to 75.0. Only human transcription factors are listed. Nucleotide codes: M = A or C, N = A, C, G or T, R = A or G, W = A or T, Y = C or T.

**Figure 4:**
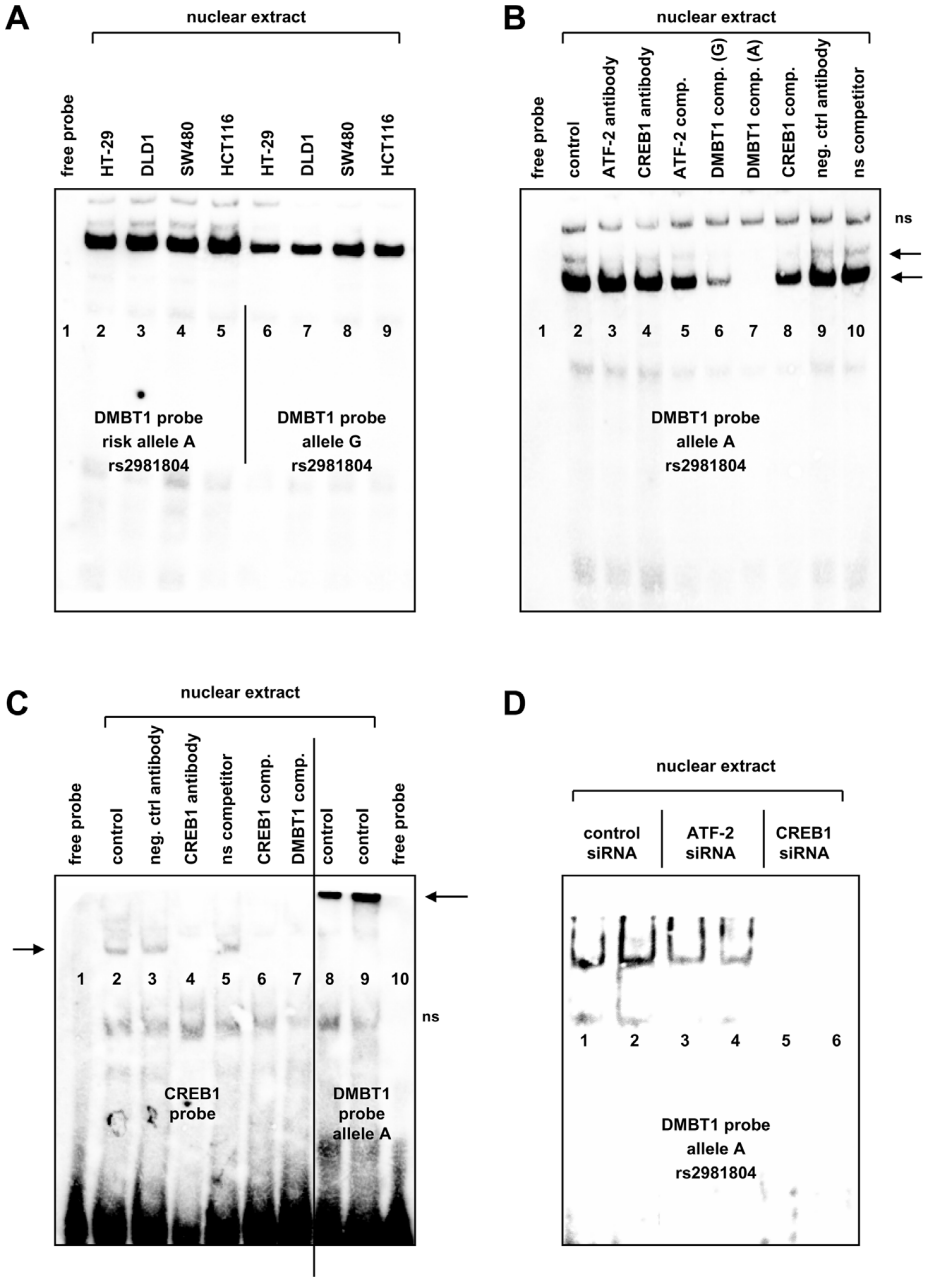
Variants in rs2981804 alter binding of the transcription factors CREB1 and ATF-2 to the respective genomic DNA sequence. (A) EMSA analysis was performed with biotinylated probes and nuclear extracts from the intestinal epithelial cell lines HT-29 (lanes 2, 6), DLD-1 (lanes 3, 7), SW480 (lanes 4, 8) and HCT116 (lanes 5, 9). A DNA probe containing the IBD risk allele A of rs2981804 is much stronger bound by nuclear proteins than a probe with the G allele. Lane 1 contains the DNA probe only with no nuclear extract added. (B) Two specific protein complexes binding to the DMBT1 probe can be detected. The addition of a ATF-2 (lane 3) or CREB1 (lane 4) antibody to the EMSA binding reactions inhibit protein binding to the DMBT1 probe with the risk allele A while a non-specific isotype control antibody had no effect (lane 9). Addition of 50-fold excess of unlabelled CREB1, ATF-2 or DMBT1 probe (G allele) reduced protein binding (lanes 5, 6, 8). A DMBT1 probe with the A allele completely abolished protein binding (lane 7) while an unlabelled non-specific DNA probe did not inhibit DNA-protein complex formation confirming specificity of binding (lane 10); ns = non-specific. (C) In EMSAs with a labelled CREB1 consensus probe (lanes 1–7), the addition of 50-fold excess of unlabelled DMBT1 probe inhibited protein binding (lane 7) as well as did unlabelled CREB1 probe or a CREB1 antibody (lanes 4, 6). The protein complex bound to a labelled DMBT1 probe (lanes 8–9) is migrating slower (and therefore larger) than that of the CREB1 probe. (D) Silencing of ATF-2 or CREB1 expression diminished protein binding to the DMBT1 probe. DLD-1 cells were transfected with siRNA against CREB1, ATF-2 or a non-specific control siRNA 48 h prior to nuclear protein isolation. EMSA was performed as in Fig. 2A with a DMBT1 probe containing the A allele. While expression silencing of CREB1 completely abolished protein binding to the DMBT1 probe, silencing of ATF-2 had a weaker but still detectable effect.

In EMSA experiments with probes containing either the IBD risk allele A or the protective allele G of rs2981804 and the surrounding genomic sequences, nuclear extracts from IEC lines HT-29, DLD-1, HCT116 and SW480 bound strongly to the probe containing the risk allele A. The probe with the IBD-protective allele G showed clearly weaker protein binding (Fig. 4A). To analyze which proteins were bound to the probes, antibodies against phosphorylated cAMP responsive element binding protein 1 (CREB1) or activating transcription factor 2 (ATF-2) were incubated together with the DNA binding reaction with nuclear extracts from DLD-1 cells. For both transcription factors, higher binding scores were predicted for the A allele in comparison to the G allele (table 6). The addition of an ATF-2 or a CREB1 antibody diminished but did not abolish DNA binding of protein to the DMBT1 probe (Fig. 4B, lanes 3, 4) as well as did unlabelled competitor probes with binding sequences for ATF-2 or CREB1 (Fig. 4B, lanes 5, 8). Addition of 50-fold excess of an unlabelled DMBT1 probe with the A allele completely inhibited binding to the labelled probe while a DMBT1 probe with the G allele had a weaker effect (Fig. 4B, lanes 6, 7). Control reactions (Fig. 4B, lanes 9, 10) with a non-specific antibody or a non-specific DNA probe which did not inhibit specific DNA-protein complex formation demonstrated that two specific protein complexes with two distinct sizes are bound to the DMBT1 probe (Fig. 4B, see arrows).

Next, we performed EMSA experiments using a labelled CREB1 consensus probe. CREB1 protein binding was proven by a CREB1-specific antibody (Fig. 4C, lane 4). Excess of unlabelled DMBT1 probe was able to repress protein binding to the CREB1 probe (Fig. 4C, lane 7). Moreover, on the same gel, binding reactions with a labelled DMBT1 probe were included (Fig. 4C, lanes 8–10). A more slowly migrating (and therefore larger) protein complex bound to the DMBT1 probe that had much stronger signal intensity (in comparison to the CREB1 probe) could be observed.

Then we transfected DLD-1 cells with a non-specific control siRNA or siRNA against CREB1 and ATF-2 48 hours prior to protein isolation. SiRNA-mediated down-regulation of CREB1 resulted in a nearly complete loss of protein binding to the DMBT1 probe with the risk allele (Fig. 4D). Down-regulation of ATF-2 also resulted in a slightly decreased protein binding to the DMBT probe (Fig. 4D). Silencing of CREB1 and ATF-2 by the respective siRNAs was confirmed by in western blot experiments (Fig. S4A, B). Together, our data suggest that CREB1 and ATF-2 are essential parts of a larger multi-protein complex that binds strongly to the DMBT1 probe containing the IBD risk allele of rs2981804 but substantially less to the sequence with the protective allele.

### The Th17 Cytokine IL-22 Stimulates DMBT1 Expression in Intestinal Epithelial Cells Dependent on the Transcription Factors CREB1 and ATF-2

**Figure 5:**
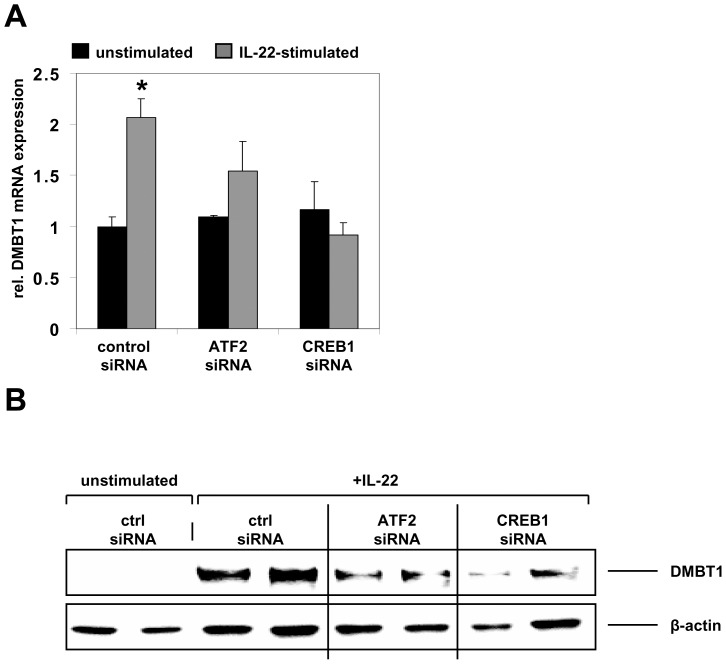
IL-22-induced DMBT1 expression depends on the transcription factors CREB1 and ATF-2. (A) Silencing of CREB1 by siRNA transfection completely abolished IL-22-induced DMBT1 mRNA expression while ATF2 silencing had a weaker negative effect. Data are from three independent experiments. *p = 0.01 vs. control unstimulated. (B) DMBT1 protein expression is induced in IEC by treatment with IL-22 for 48 h. Knockdown of ATF-2 or CREB1 expression prior to stimulation diminished IL-22-induced DMBT1 expression.

Next, we aimed to confirm that ATF2 and CREB1 are involved in the transcriptional regulation of DMBT1. As we have shown that IL-22 is an inducer of DMBT1 gene expression in IEC (Fig. 2A), we next analyzed DMBT1 expression in IL-22-stimulated DLD-1 cells in which expression of CREB1 or ATF-2 was silenced by siRNA transfection. In control siRNA-transfected cells, expression of DMBT1 was significantly up-regulated by IL-22 after 6 hours of stimulation (p = 0.01; Fig. 5A). In DLD-1 cells transfected with CREB1 siRNA, no increase in DMBT expression was observed following IL-22 stimulation (Fig. 5A). Silencing of ATF-2 prior to IL-22 stimulation had an intermediate effect as IL-22-induced DMBT1 up-regulation was still detectable but weaker than that of control siRNA-transfected cells stimulated with IL-22 (Fig. 5A). Western blot experiments with a DMBT1 antibody confirmed that the IL-22-induced DMBT1 protein expression is inhibited by silencing of CREB1 or ATF-2 expression (Fig. 5B).

### The Genomic Region Comprising *DMBT1* SNP rs2981804 Differentially Influences Promoter Activity

To analyze whether the genomic region comprising the *DMBT1* SNP rs2981804 directly influences gene transcription *in vitro*, we cloned a PCR-amplified DMBT1 fragment with either the rs2981804 A or G allele into two luciferase reporter vectors containing either a minimal promoter (minP) or a strong SV40 promoter/enhancer as described in the methods and methods S1 section. The inserts were cloned either immediately upstream, 2 kb downstream or up- and downstream of the promoter-luciferase gene region to analyze potential position-dependent short-range and long-range effects on gene expression.

**Figure 6:**
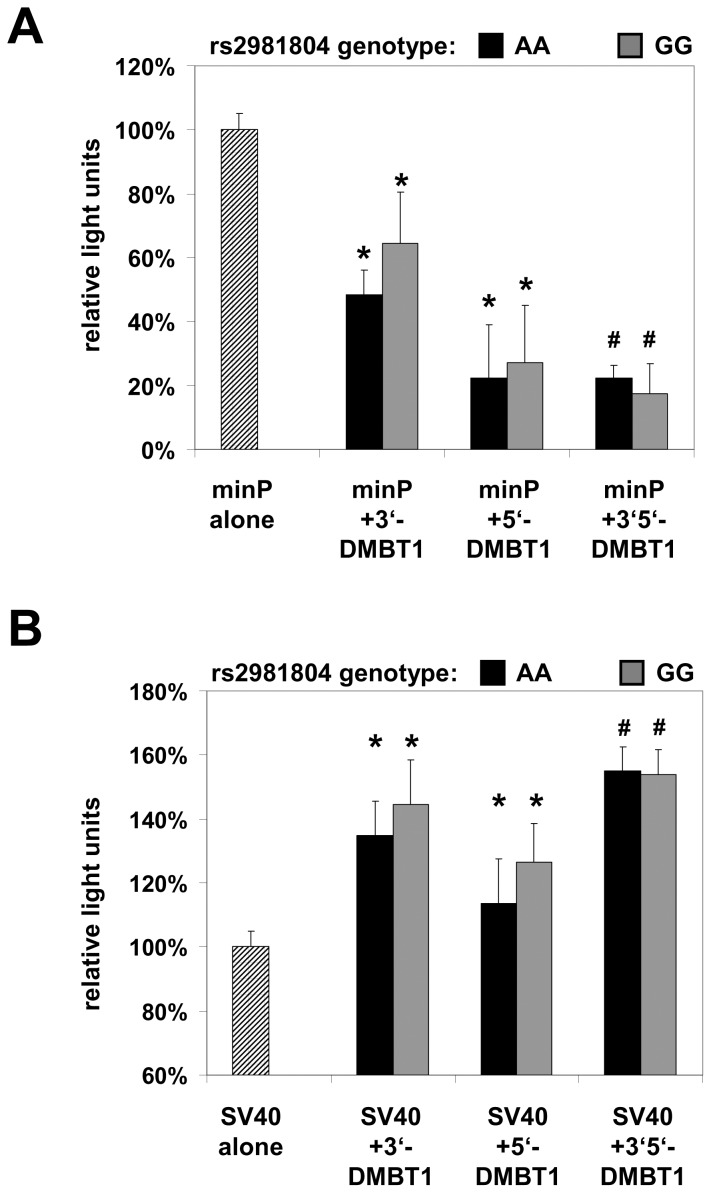
The genomic region comprising *DMBT1* SNP rs2981804 differentially influences promoter activity. Luciferase assays were performed in DLD-1 cells with *DMBT1* inserts comprising SNP rs2981804 A or G alleles. When cloned into a vector with weak minimal promoter (A), the *DMBT1* insert repressed luciferase expression while together with a strong SV40 promoter, it increases gene expression (B). *p<0.01; ^#^p<0.001 vs. empty vector.

Luciferase assay in transfected DLD-1 cells revealed that in the minP vector with low basal expression, the *DMBT1* region suppresses gene transcription, especially when cloned both up- and downstream of the promoter-luciferase gene (Fig. 6A). When cloned into a strong SV40 promoter-driven luciferase expression vector, the *DMBT1* fragment was able to further increase luciferase gene expression (Fig. 6B). However, there were no significant differences between the A and the G allele of rs2981804. Similar results were obtained in HT-29 cells (data not shown).

### Intestinal DMBT1 Expression is Lower in Homozygous Carriers of the *DMBT1* rs2981804 IBD Risk Allele

**Figure 7:**
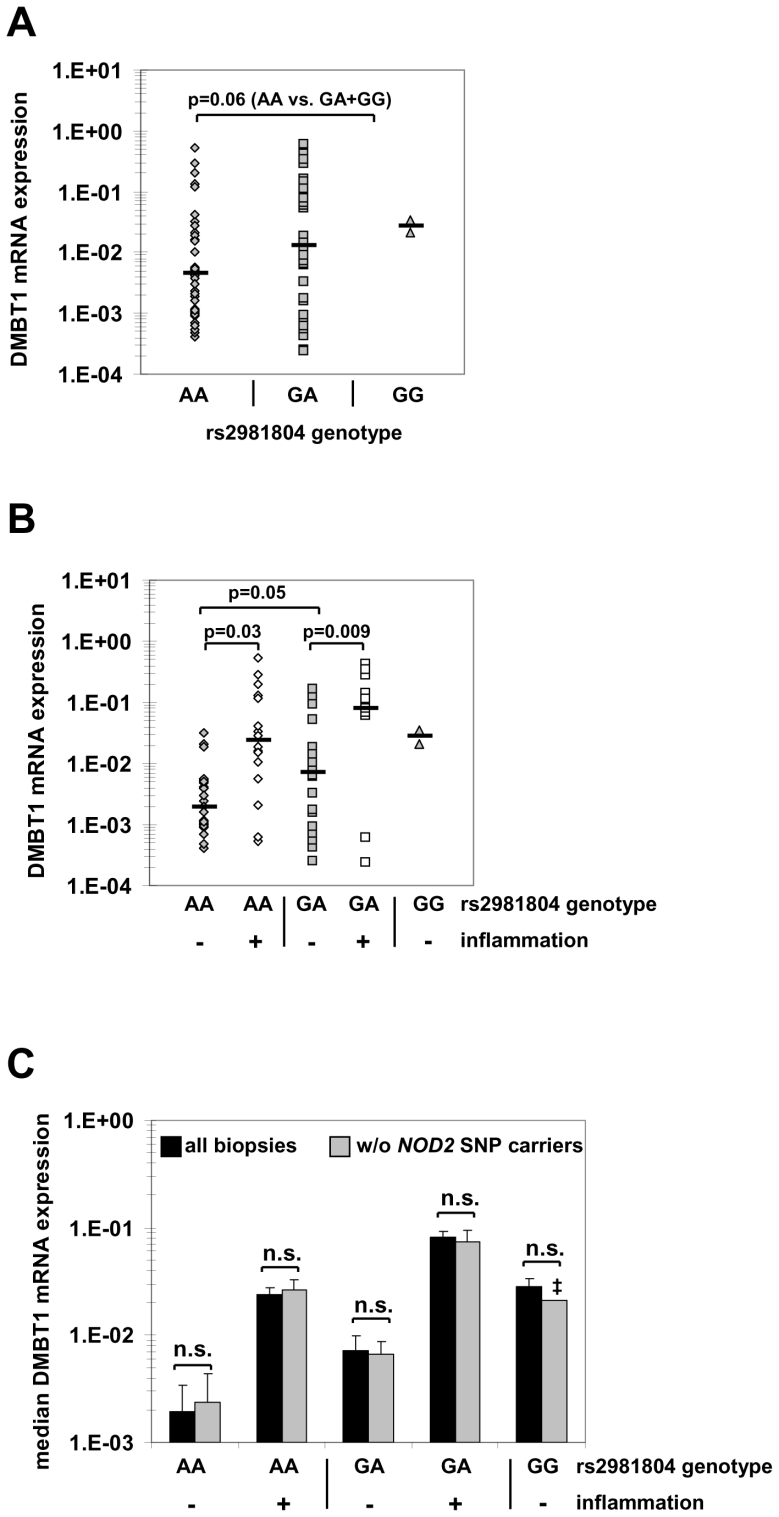
The CD risk allele of *DMBT1* SNP rs2981804 is associated with lower colonic DMBT1 expression. (A) Colonic DMBT1 mRNA expression was determined by quantitative PCR and was normalized to β-actin expression in the respective samples (n = 75 biopsies from 27 CD patients). There was a trend towards higher DMBT1 expression in carriers of the G allele (GA+GG) in comparison to AA carriers (p = 0.06). Each dot represents one biopsy and the black horizontal bar is the median of each group. (B) When biopsies were subdivided according to inflammation status, homozygous carriers of the AA risk allele of SNP rs2981804 had lower basal and inflammation-induced DMBT1 expression levels in comparison to carriers of the G allele (p = 0.03 AA vs. GA+GG). (C) DMBT1 mRNA expression is independent of *NOD2* genotype status. DMBT1 mRNA expression was determined in biopsies from CD patients irrespective of *NOD2* genotype (n = 75; black bars) or excluding biopsies from *NOD2* SNP carriers (n = 45 *NOD2* wild-type carriers; grey bars). Data are presented as median DMBT1 expression in the respective groups. There were no significant differences in DMBT1 expression between all biopsies and *NOD2* wild-type carriers for the different DMBT1 rs2981804 genotypes. n.s. = not significant; ‡: no standard deviation is given for GG *NOD2* wild-type carriers as this group comprised only one biopsy.

To determine whether rs2981804 is associated with differential DMBT1 expression in CD patients, DMBT1 mRNA expression levels measured in human intestinal biopsies (Fig. 3B) were subdivided according to the rs2981804 genotype [AA: n = 11 patients, 36 biopsies, 21 not inflamed, 15 inflamed; GA: n = 14 patients, 37 biopsies, 22 not inflamed, 15 inflamed; GG: n = 2 patients, 2 biopsies (both not inflamed)]. Overall, a trend towards higher DMBT1 expression in carriers of the protective G allele was observed (Fig. 7A, p = 0.06, AA vs. GA+GG). An inflammation-induced increase in DMBT1 expression was observed for both rs2981804 AA and GA carriers (Fig. 7B). However, basal DMBT1 expression was lower in homozygous carriers of the IBD risk allele (AA) than in GA and GG carriers (p = 0.052 AA vs. GA, p = 0.03 AA vs. GA+GG) (Fig. 7B). Similar results were obtained when *NOD2* SNP carriers (see table 2) were excluded from analysis (Fig. 7C).

## Discussion

In this study, we performed a detailed functional analysis of the *DMBT1* gene. First, we investigated the effect of the Th17 cytokine IL-22 on DMBT1 expression. In these detailed experiments, we confirmed DMBT1 as a target gene of the cytokine IL-22. Our results are supported by a recent study [22]. IL-22 is produced by IL-23R-expressing Th17 cells [36] and we have recently shown that variants in *IL23R* influencing the CD risk are associated with differential IL-22 expression [34]. The same *IL23R* variants are also associated with differential intestinal DMBT1 expression suggesting a functional link between *IL23R* genotype, IL-22 expression and DMBT1 expression. A very recent study demonstrated that DMBT1 expression in intestinal inflammation is influenced by antibody treatment against IL-23R or IL-23 [24], further supporting this hypothesis. Interestingly, we recently demonstrated that the clinical response to infliximab, an anti-TNF-α antibody used for treatment of IBD, is associated with certain *IL23R* genotypes in UC patients [41].

In this study, we show that several previously not analyzed *DMBT1* SNPs and their haplotypes are associated with the susceptibility to IBD. The two most strongly IBD-associated *DMBT1* SNPs were the non-coding SNPs rs2981745 and rs2981804 for which the minor allele frequencies were significantly higher in CD and UC patients compared to controls. Associations remained significant even after conditioning the analysis on the most strongly-associated SNP for each disease. Considering the weak LD between both SNPs (r^2^ = 0.24, 0.18 and 0.17 for CD, UC, and controls, respectively), our data suggest largely independent associations with IBD susceptibility. We also demonstrated for a large number of *DMBT1* haplotypes highly significant associations with CD and UC, with *P*-values as low as 10^−18^. A recent study demonstrated that a deletion variant in the repetitive region of *DMBT1* encoding the SRCR domains is associated with CD [20]. Although it can not completely be excluded that the SNPs analyzed in our study are in LD with this variant, it is rather unlikely. SNP rs2277244 analyzed in our study is localized within the repetitive SRCR genomic region and linkage analysis proved that this SNP is not in LD with any other SNP examined here (r^2^≤0.02) suggesting that the SNPs analyzed in our study are associated with IBD susceptibility independent of the SRCR region. However, it has to be mentioned that our association results need to be confirmed by replication in other large case-control cohorts for both CD and UC. In recent GWAS studies and meta-analyses, the *DMBT1* gene region did not appear as major risk factor for IBD [9], [32], [42], [43]. One reason might be ethnic differences between study population as it has been described for other genes such as *PHOX2B*, *NCF4* and *FAM92B* or *DLG5* [44], [45], [46], [47]. Another fact that might contribute to that lack is the poor coverage of the *DMBT1* gene region on the first available GWAS chips, likely due to the many repetitive genomic regions within the gene. Moreover, even though the most recent and so far largest GWAS meta-analysis identified 163 IBD-associated variants, these genes explain only a small part of the IBD risk variation observed. Rare haplotypes conferring a highly significant IBD risk (as we have observed in our study) might be missed.

Interestingly, a very recent study by Dinu et al. demonstrated that a considerable number of genes or chromosomal regions contribute to CD risk through SNP-SNP interactions [48]. Many of those genes were not identified in recent GWAS analyses including more than 20,000 patients and controls as these studies analyzed only single SNP associations [48]. Remarkably, Dinu et al. identified several so far unknown CD risk genes (*FGFR2*, *FOXI2*, *GLRX3*) on chromosome 10 q26, surrounding the chromosomal region that also harbours the *DMBT1* gene [48].

Interestingly, both most strongly IBD-associated *DMBT* SNPs in our study (rs2981745 and rs2981804) are located in non-coding genomic regions, thereby not altering DMBT1 protein structure or function. Therefore, we hypothesized that these SNPs might be located within recognition sequences of transcription factors. For the first time, we demonstrated that the transcription factors CREB1 and ATF-2 differentially bind to DNA probes containing either the IBD risk allele A or the protective G allele of SNP rs2981804. Moreover, we show that CREB1 and ATF-2 are involved in the transcriptional regulation of IL-22-induced *DMBT1* expression. We show in our study that the IBD risk allele of rs2981804 is associated with a lower DMBT1 gene expression in colonic tissue from CD patients identifying for the first time a link between rs2981804 alleles and DMBT1 gene expression. These results were independent of the *NOD2* genotype.

Our luciferase assays revealed that the genomic region comprising SNP rs2981804 can act either as a transcriptional activator or repressor, depending on the promoter context. Given that enhancers or repressors usually do not induce or repress gene expression directly but interact with promoter-specific transcription factors, such differences can be explained. Similar results have been described for example for the c-myc enhancer [49]. There is the possibility that the respective genomic region comprising *DMBT1* SNP rs2981804 not solely influences DMBT1 expression. As enhancers/repressors can be located several hundred kb away from genes which they regulate [50], the expression of other genes located within this chromosomal region might be influenced as well. Moreover, CREB1 and ATF-2 are transcription factor that are ubiquitously expressed and are involved in the regulation of many genes and cellular processes such as immunity, cell proliferation, differentiation, and cell survival [51]. Therefore, further analysis is necessary to determine whether the genomic region comprising *DMBT1* SNP rs2981804 influences expression of other genes *in vivo*. Moreover, the differential influence of this SNP on DMBT1 expression, especially in the presence of different transcription factors and in the context of different cytokine environments that have a strong influence on DMBT1 expression [12], [22], [23], should be aims of future analyses.

In conclusion, we demonstrated novel significant associations of *DMBT1* variants and haplotypes with the susceptibility to CD. Moreover, we provide for the first time functional evidence that the non-coding *DMBT1* SNP rs2981804 modifies the binding sites for the transcription factors CREB1 and ATF-2. This *DMBT1* SNP is associated with decreased overall DMBT1 expression in the colon thereby probably contributing to increased CD susceptibility. As *DMBT1* encodes - like *NOD2* - an antibacterial pattern recognition receptor [11], our results support the hypothesis that a dysregulated antibacterial response of the innate immune system might contribute to the pathogenesis of CD.

## Supporting Information

Figure S1 **Overview of the DMBT1 luciferase reporter constructs in a vector with a minimal promoter-driven luciferase expression.** For cloning details, see supplementary methods. AmpR, ampicillin resistance gene; luc2, luciferase gene; ori, origin of replication; minP, minimal promoter.
(DOC)

Figure S2 **Overview of the used DMBT1 luciferase reporter constructs with an SV40 promoter driven luciferase expression.** For cloning details, see supplementary methods. AmpR, ampicillin resistance gene; luc2, luciferase gene; ori, origin of replication.
(DOC)

Figure S3 ***IL23R*** **CD risk-increasing variants (OR>1) are associated with higher DMBT1 expression in minor allele carriers (minor vs. WT>1) independent of inflammation status.** Intestinal DMBT1 expression and *IL23R* genotypes were determined in 75 biopsies from 27 CD patients. DMBT1 expression is presented as a quotient derived from dividing expression in minor allele carriers of the respective *IL23R* SNP by the expression in WT carriers. The ORs for the respective SNPs were available from a previous study [8].
(DOC)

Figure S4 **Western blot analysis of siRNA-transfected nuclear extracts shows effective silencing of CREB1 (A) and ATF-2 (B) protein expression.** The nuclear matrix protein p84 was used as loading control.
(DOC)

Table S1 **Primers used for cloning of the DMBT1 fragment containing SNP rs2981804 and for analysis of luciferase reporter constructs.** The respective restriction enzyme recognition sites are underlined. All primers sequences are given in 5′-3′ orientation. PCR product sizes for vectors are given for empty vectors without inserts.
(DOC)

Table S2 **Primer sequences (F: forward primer, R: reverse Primer), and FRET probe sequences used for genotyping** ***DMBT1*** **variants.** Note: FL: Fluorescein, LC610: LightCycler-Red 610; LC640: LightCycler-Red 640, LC670: LightCycler-Red 670. The polymorphic position within the sensor probe is underlined. A phosphate is linked to the 3′-end of the acceptor probe to prevent elongation by the DNA polymerase in the PCR.
(DOC)

Table S3 **Primer sequences used for the sequence analysis of** ***DMBT1*** **variants.**
(DOC)

Table S4 **Hardy Weinberg analysis of the control panel for all** ***DMBT1*** **SNPs.** *P*-values are corrected for multiple testing using the Bonferroni method (n = 7 tests; significant *P*-value threshold 0.05/7 = 0.007).
(DOC)

Table S5 **Association results of** ***DMBT1*** **gene markers in the CD discovery and CD replication case-control panels.** Minor allele frequencies (MAF), allelic test *P*-values (1 degree of freedom), and odds ratios (OR; shown for the minor allele) with 95% confidence intervals (CI) are depicted for both CD case-control cohorts. *P*-values <0.05 are highlighted in **bold** and *P*-values robust to multiple testing (P<0.0036) are highlighted in ***bold italic***. Suggestive p-values (p<0.10) are given in *Italic* fonts.
(DOC)

Table S6 **Linkage disequilibrium (LD) matrix for** ***DMBT1*** **SNPs in CD and UC patients and controls.** Values are given as D′/r^2^.
(DOC)

Table S7 **Association results of** ***DMBT1*** **gene variants with UC.** Risk allele frequencies (RAF), allelic test *P*-values (1 degree of freedom), and odds ratios (OR, shown for the riskallele) with 95% confidence intervals (CI) are depicted for the UC case-control panel. *P*-values <0.05 are highlighted in **bold** and *P*-values robust to multiple testing (P<0.0036) are highlighted in ***bold italic***. P-values are based on 10,000,000 permutations.
(DOC)

Table S8 **Association between** ***DMBT1*** **rs2981745 genotypes and CD disease characteristics in the subcohort of the Munich IBD center (n = 628) for which detailed phenotypic data based on the Montreal classification were available.** For each variable, the number of patients included is given. P_T_, *P*-value for testing for differences between carriers and non-carriers of the T allele. OR_T_: corresponding odds ratios and 95% confidence intervals (95% CI). For age at diagnosis, *P*-values are given based on a median split. Significant *P*-values are depicted in bold. However, after Bonferroni correction for multiple testing, significance was lost. ^1^Disease behaviour was defined according to the Montreal classification. A stricturing disease phenotype was defined as presence of stenosis without penetrating disease. The diagnosis of stenosis was made surgically, endoscopically, or radiologically (using MRI enteroclysis). ^2^Immunosuppressive agents included azathioprine, 6-mercaptopurine, 6-thioguanin, methotrexate, infliximab and/or adalimumab.^ 3^Only surgery related to CD-specific problems (e.g. fistulectomy, colectomy, ileostomy) was included.
(DOC)

Table S9 **Association between** ***DMBT1*** **rs2981745 genotypes and UC disease characteristics in the subcohort of the Munich IBD center (n = 283) for which detailed phenotypic data based on the Montreal classification were available.** For each variable, the number of patients included is given. P_T_, *P*-value for testing for differences between carriers and non-carriers of the T allele. OR_T_: corresponding odds ratios and 95% confidence intervals (95% CI). For age at diagnosis, age and BMI *P*-values are given based on a median split. Significant association is highlighted in bold. However, after Bonferroni correction for multiple testing, this significance was lost.
(DOC)

Table S10 **Association between** ***DMBT1*** **rs2981804 genotypes and CD disease characteristics in the subcohort of the Munich IBD center (n = 626) for which detailed phenotypic data based on the Montreal classification were available.** For each variable, the number of patients included is given. P_G_, *P*-value for testing for differences between carriers and non-carriers of the G allele. OR_G_: corresponding odds ratios and 95% confidence intervals (95% CI). For age at diagnosis *P*-values are given based on a median split. ^1^Disease behaviour was defined according to the Montreal classification. A stricturing disease phenotype was defined as presence of stenosis without penetrating disease. The diagnosis of stenosis was made surgically, endoscopically, or radiologically (using MRI enteroclysis). ^2^Immunosuppressive agents included azathioprine, 6-mercaptopurine, 6-thioguanin, methotrexate, infliximab and/or adalimumab. ^3^Only surgery related to CD-specific problems (e.g. fistulectomy, colectomy, ileostomy) was included.
(DOC)

Table S11 ***DMBT1*** **gene markers in CD – Haplotype frequencies (HF),** ***P*** **-values, and odds ratios (OR) with 95% confidence intervals (CI).** *P-*values for individual haplotypes are presented for all haplotypes with a frequency of at least 1% in the whole sample and with an omnibus haplotype *P-*value <0.05. Significant *P-*values (<0.05) are highlighted in **bold** and significant *P*-values robust to multiple testing (*P*<2.5×10^−3^ *for omnibus P*-values, *P*<4.8×10^−4^ for detailed haplotype *P-*values) are highlighted in ***bold italic***.
(DOC)

Table S12 ***DMBT1*** **gene markers in UC – Haplotype frequencies (HF),** ***P*** **-values, and odds ratios (OR) with 95% confidence intervals (CI).** *P-*values for individual haplotypes are presented for all haplotypes with a frequency of at least 1% in the whole sample and with an omnibus haplotype *P-*value <0.05. Significant *P-*values (<0.05) are highlighted in **bold** and significant *P*-values robust to multiple testing (*P*<2.5×10^−3^ *for omnibus P*-values, *P*<4.810^−4^ for detailed haplotype *P-*values) are highlighted in ***bold italic***.
(DOC)

Table S13 **Analysis for epistasis between SNPs rs2981745, rs2981778, rs11523871 = p.Pro42Thr, rs3013236 = p.Leu54Ser, rs2981804, rs2277244 = p.His585Tyr, rs1052715 = p.Pro1707Pro within the** ***DMBT1*** **gene and the SNP rs151181 in the** ***IL27*** **gene region regarding CD/UC susceptibility.** All *P*-values given are uncorrected for multiple comparisons.
(DOC)

Table S14 **Analysis for epistasis between SNPs rs2066844 = p.Arg702Trp, rs2066845 = p.Gly908Arg and rs2066847 = p.Leu1007fsX1008 in the** ***NOD2*** **gene and the SNPs** ***DMBT1*** **rs2981745 and rs2981804 within the** ***DMBT1*** **gene regarding CD/UC susceptibility.** All *P*-values given are uncorrected for multiple comparisons.
(DOC)

Table S15 **Analysis for epistasis between SNPs rs1004819, rs7517847, rs10489629, rs2201841, rs11465804, rs11209026 = p.Arg381Gln, rs1343151, rs10889677, rs11209032, rs1495965 in the** ***IL23R*** **gene and the SNPs** ***DMBT1*** **rs2981745 and rs2981804 within the** ***DMBT1*** **gene regarding CD/UC susceptibility.** All *P*-values given are uncorrected for multiple comparisons. Nominal significant *P-*values are indicated in bold.
(DOC)

Methods S1 **Supplementary methods.**
(DOC)
