# Supplementary material for: Intestinal DMBT1 Expression Is Modulated by Crohn’s Disease-Associated IL23R Variants and by a DMBT1 Variant Which Influences Binding of the Transcription Factors CREB1 and ATF-2
Source: PLoS One. 2013 Nov 5;8(11):e77773. doi: 10.1371/journal.pone.0077773 (PMC3818382; doi:10.1371/journal.pone.0077773)
Supplement: Table S1 — Primers used for cloning of the DMBT1 fragment containing SNP rs2981804 and for analysis of luciferase reporter constructs. The respective restriction enzyme recognition sites are underlined. All primers sequences are given in 5′-3′ orientation. PCR product sizes for vectors are given for empty vectors without inserts. (DOC) [file pone.0077773.s005.doc]

|  | **Primer sequences** | **expected size**  **of PCR product (bp)** |
| --- | --- | --- |
| rs2981804 amplification Kpn-Xho | F: AGAGGGTACCGCGTCTCCAAGGAGTTCTCA  R: AGAGCTCGAGGCCAGAATCCCAGGATGTA | 364 |
| rs2981804 amplification Bam-Xho | F: AGAGGGATCCGCGTCTCCAAGGAGTTCTCA  R: AGAGCTCGAGGCCAGAATCCCAGGATGTA | 364 |
| primer for pGL4.23 and pGL4.13  5’ insert | F: AGTGCAGGTGCCAGAACATT  R: CCGTCTTCGAGTGGGTAGAA | 223 (pGL4.23)  594 (pGL4.13) |
| primer for pGL4.23 and pGL4.13  3’ insert | F: GGGAGGTGTGGGAGGTTTT  R: GAGTCAGTGAGCGAGGAAGC | 222 |

**Table S1.** **Primers used for cloning of the DMBT1 fragment containing SNP rs2981804 and for analysis of luciferase reporter constructs.** The respective restriction enzyme recognition sites are underlined. All primers sequences are given in 5’-3’ orientation. PCR product sizes for vectors are given for empty vectors without inserts.
