# Supplementary material for: Intestinal DMBT1 Expression Is Modulated by Crohn’s Disease-Associated IL23R Variants and by a DMBT1 Variant Which Influences Binding of the Transcription Factors CREB1 and ATF-2
Source: PLoS One. 2013 Nov 5;8(11):e77773. doi: 10.1371/journal.pone.0077773 (PMC3818382; doi:10.1371/journal.pone.0077773)
Supplement: Table S2 — Primer sequences (F: forward primer, R: reverse Primer), and FRET probe sequences used for genotyping DMBT1 variants. Note: FL: Fluorescein, LC610: LightCycler-Red 610; LC640: LightCycler-Red 640, LC670: LightCycler-Red 670. The polymorphic position within the sensor probe is underlined. A phosphate is linked to the 3′-end of the acceptor probe to prevent elongation by the DNA polymerase in the PCR. (DOC) [file pone.0077773.s006.doc]

| **Polymorphism** | **Primer sequences** | **FRET probe sequences** |
| --- | --- | --- |
| rs2981745 | F: CAATTGAATAGAAAATCCTGGACATT  R: TTTCCACATATTAGCCATCGAC | CAGCAGCAGAAATATACCACCC-FL  LC610-AGAGGACACACCTCCTTTTAGCTAGGTACC |
| rs2981778 | F: CCCTTGACTTGCATCCCTAC  R: GGTTCTCCAGGGGGAAA | GGATCTTAAGTATCCCTGTTCTTCC-FL  LC610-TGTTCTCTCTTTGCCCTCTTCTCATTCT |
| rs11523871 | F: CAATGAGCTCTTCCTTTTCCAC  R: GAATGCCAGAGTTGCTAGAAGTATG | CTTCTGCTACAGTTGTATCCAAGG-FL  LC640-CACCTCCGAGGGAATCAGTGAAG |
| rs3013236 | F: CCCAAACAAGGGCTACCATCAAT  R: CCAGCACCCTACCTTCTATGCC | LC640-GGGTCAACTCCGAGGGAA  AGACGTTACCTTCTGCTACAGTTGACTCC-FL |
| rs2981804 | F: TGATGCCCATGAGCTGAA  R: AGCTGTGCTCATCCGTG | LC670-GTCCTAGCAATTTGGCTACGTTA  AGGGCAAGGGCAAGGATGC-FL |
| rs2277244 | F: TGAGTGAGGAGATCAGAATGTAA  R: ACACCAATGATGCCAATG | CAGCGTCTTCACTATAGCCAC-FL  LC640-GTTGTGGGAGAGCCAGCCA |
| rs1052715 | F: GGCTACTGTTCTCTTCCAGA  R: CATTTTACAACGCAGGTACA | ATGCGAAGAGACGGCGAG-FL  LC670-AGTAGGGTCCGTAGGTGTCATCCCTCA |

**Table S2. Primer sequences (F: forward primer, R: reverse Primer), and FRET probe sequences used for genotyping *DMBT1* variants.** Note: FL: Fluorescein, LC610: LightCycler-Red 610; LC640: LightCycler-Red 640, LC670: LightCycler-Red 670. The polymorphic position within the sensor probe is underlined. A phosphate is linked to the 3'-end of the acceptor probe to prevent elongation by the DNA polymerase in the PCR.
