# Supplementary material for: Intestinal DMBT1 Expression Is Modulated by Crohn’s Disease-Associated IL23R Variants and by a DMBT1 Variant Which Influences Binding of the Transcription Factors CREB1 and ATF-2
Source: PLoS One. 2013 Nov 5;8(11):e77773. doi: 10.1371/journal.pone.0077773 (PMC3818382; doi:10.1371/journal.pone.0077773)
Supplement: Table S3 — Primer sequences used for the sequence analysis of DMBT1 variants. (DOC) [file pone.0077773.s007.doc]

| **Polymorphism** | **Primer sequences** |
| --- | --- |
| rs2981745 | ATTTCAAGGATGACTGTGGAGAT  TTTCCACATATTAGCCATCGAC |
| rs2981778 | CCCTTGACTTGCATCCCTAC  GGTCTTCTGGAAAGTTCTCAGAGTAC |
| rs11523871 | GCTGAGGAAGCCAGGGAC  GAATGCCAGAGTTGCTAGAAGTATG |
| rs3013236 | AGGTTGCCCTTAGGATCTGTGTTT  CCAGCACCCTACCTTCTATGCC |
| rs2981804 | TGATGCCCATGAGCTGAA  AGCTGTGCTCATCCGTG |
| rs2277244 | GGGTTGTTTGAGTTTGGTC  GTCCTGGATGATGTGCG |
| rs1052715 | AACTGAGTCATGAAGGAAGAATC  GGTCATACGCTCTGCACA |

**Table S3. Primer sequences used for the sequence analysis of *DMBT1* variants.**
