# Supplementary material for: Intestinal DMBT1 Expression Is Modulated by Crohn’s Disease-Associated IL23R Variants and by a DMBT1 Variant Which Influences Binding of the Transcription Factors CREB1 and ATF-2
Source: PLoS One. 2013 Nov 5;8(11):e77773. doi: 10.1371/journal.pone.0077773 (PMC3818382; doi:10.1371/journal.pone.0077773)
Supplement: Table S4 — Hardy Weinberg analysis of the control panel for all DMBT1 SNPs. P-values are corrected for multiple testing using the Bonferroni method (n = 7 tests; significant P-value threshold 0.05/7 = 0.007). (DOC) [file pone.0077773.s008.doc]

| **SNP** | **all controls**  ***P*-value** | **male**  ***P*-value** | **female**  ***P*-value** |
| --- | --- | --- | --- |
| **rs2981745** | 0.490 | 0.140 | 1.000 |
| **rs2981778** | 1.000 | 0.114 | 1.000 |
| **rs11523871 =p.Pro42Thr** | 1.000 | 0.266 | 1.000 |
| **rs3013236 =p.Leu54Ser** | 1.000 | 0.231 | 1.000 |
| **rs2981804** | 0.168 | 0.063 | 1.000 |
| **rs2277244 =p.His585Tyr** | 1.000 | 1.000 | 1.000 |
| **rs1052715 =p.Pro1707Pro** | 0.070 | 0.336 | 0.714 |

**Table S4.** **Hardy Weinberg analysis of the control panel for all *DMBT1* SNPs analyzed in this study.** *P*-values are corrected for multiple testing using the Bonferroni method (n=7 tests; significant *P*-value threshold 0.05/7=0.007).
