# Supplementary material for: Intestinal DMBT1 Expression Is Modulated by Crohn’s Disease-Associated IL23R Variants and by a DMBT1 Variant Which Influences Binding of the Transcription Factors CREB1 and ATF-2
Source: PLoS One. 2013 Nov 5;8(11):e77773. doi: 10.1371/journal.pone.0077773 (PMC3818382; doi:10.1371/journal.pone.0077773)
Supplement: Table S5 — Association results of DMBT1 gene markers in the CD discovery and CD replication case-control panels. Minor allele frequencies (MAF), allelic test P-values (1 degree of freedom), and odds ratios (OR; shown for the minor allele) with 95% confidence intervals (CI) are depicted for both CD case-control cohorts. P-values <0.05 are highlighted in bold and P-values robust to multiple testing (P<0.0036) are highlighted in bold italic. Suggestive p-values (p<0.10) are given in Italic fonts. (DOC) [file pone.0077773.s009.doc]

| **SNP** | **Minor allele** | **CD discovery panel**  n= 623 cases / 762 controls | | | | **CD replication panel**  n= 195 cases / 210 controls | | | |
| --- | --- | --- | --- | --- | --- | --- | --- | --- | --- |
|  |  | **MAF**  **CD** | **MAF**  **Controls** | ***P*-value** | **OR**  **[95 % CI]** | **MAF**  **CD** | **MAF**  **Controls** | ***P-*value** | **OR**  **[95% CI]** |
| rs2981745 | T | 0.40 | 0.33 | ***3.5×10^-5^*** | 1.39  [1.19-1.62] | 0.27 | 0.33 | *0.065* | 0.75  [0.56-1.02] |
| rs2981778 | A | 0.31 | 0.33 | 0.232 | 0.91  [0.77-1.07] | 0.27 | 0.35 | **0.026** | 0.71  [0.53-0.96] |
| rs11523871  (p.Pro42Thr) | C | 0.30 | 0.32 | 0.254 | 0.91  [0.78-1.07] | 0.27 | 0.35 | **0.028** | 0.72  [0.53-0.96] |
| rs3013236  (p.Leu54Ser ) | C | 0.30 | 0.33 | 0.153 | 0.89  [0.76-1.05] | 0.27 | 0.34 | **0.023** | 0.71  [0.52-0.95] |
| rs2981804 | A | 0.56 | 0.47 | ***1.2×10^-6^*** | 1.45  [1.25-1.69] | 0.57 | 0.50 | *0.056* | 1.31  [0.98-1.75] |
| rs2277244  (p.His585Tyr) | T | 0.03 | 0.03 | 0.613 | 0.89  [0.56-1.40] | 0.02 | 0.05 | **0.013** | 0.35  [0.15-0.83] |
| rs1052715  p.Pro1707Pro | G | 0.42 | 0.42 | 0.879 | 1.01  [0.87-1.18] | 0.43 | 0.57 | ***6.9x10^-5^*** | 0.57  [0.43-0.76] |

**Table S5. Association results of *DMBT1* gene markers in the CD discovery and CD replication case-control panels.** Minor allele frequencies (MAF), allelic test *P*-values (1 degree of freedom), and odds ratios (OR; shown for the minor allele) with 95% confidence intervals (CI) are depicted for both CD case-control cohorts. *P*-values <0.05 are highlighted in **bold** and *P*-values robust to multiple testing (P<0.0036) are highlighted in ***bold italic***. Suggestive p-values (p<0.10) are given in *Italic* fonts.
