# Supplementary material for: Intestinal DMBT1 Expression Is Modulated by Crohn’s Disease-Associated IL23R Variants and by a DMBT1 Variant Which Influences Binding of the Transcription Factors CREB1 and ATF-2
Source: PLoS One. 2013 Nov 5;8(11):e77773. doi: 10.1371/journal.pone.0077773 (PMC3818382; doi:10.1371/journal.pone.0077773)
Supplement: Table S6 — Linkage disequilibrium (LD) matrix for DMBT1 SNPs in CD and UC patients and controls. Values are given as D′/r2. (DOC) [file pone.0077773.s010.doc]

| ***DMBT1* SNPs** | **rs2981745** | **rs2981778** | **rs11523871** | **rs3013236** | **rs2981804** | **rs2277244** | **rs1052715** |
| --- | --- | --- | --- | --- | --- | --- | --- |
| **CD patients** |  |  |  |  |  |  |  |
| **rs2981745** | * | 0.87/0.54 | 0.90/0.57 | 0.86/0.52 | 0.56/0.24 | 0.49/<0.01 | 0.07/<0.01 |
| **rs2981778** | * | * | 0.94/0.88 | 1.00/0.97 | 1.00/0.55 | 1.00/0.01 | 0.03/<0.01 |
| **rs11523871** | * | * | * | 0.94/0.85 | 0.96/0.50 | 1.00/0.01 | 0.04/<0.01 |
| **rs3013236** | * | * | * | * | 1.00/0.53 | 1.00/0.01 | 0.01/<0.01 |
| **rs2981804** | * | * | * | * | * | 0.45/<0.01 | 0.28/0.08 |
| **rs2277244** | * | * | * | * | * | * | 0.71/0.02 |
| **rs1052715** | * | * | * | * | * | * | * |
| **UC patients** |  |  |  |  |  |  |  |
| **rs2981745** | * | 0.83/0.44 | 0.82/0.46 | 0.83/0.43 | 0.46/0.18 | 0.27/<0.01 | 0.09/0.01 |
| **rs2981778** | * | * | 0.97/0.87 | 1.00/0.98 | 0.99/0.53 | 0.96/0.02 | 0.03/<0.01 |
| **rs11523871** | * | * | * | 0.96/0.85 | 0.88/0.45 | 1.00/0.02 | 0.08/<0.01 |
| **rs3013236** | * | * | * | * | 0.99/0.51 | 1.00/0.02 | 0.02/<0.01 |
| **rs2981804** | * | * | * | * | * | 0.52/0.01 | 0.35/0.10 |
| **rs2277244** | * | * | * | * | * | * | 0.71/0.03 |
| **rs1052715** | ***** | ***** | ***** | ***** | ***** | ***** | ***** |

| ***DMBT1* SNPs** | **rs2981745** | **rs2981778** | **rs11523871** | **rs3013236** | **rs2981804** | **rs2277244** | **rs1052715** |
| --- | --- | --- | --- | --- | --- | --- | --- |
| **controls** |  |  |  |  |  |  |  |
| **rs2981745** | * | 0.98/0.95 | 0.98/0.96 | 0.97/0.95 | 0.63/0.17 | 1.00/0.02 | 0.15/<0.01 |
| **rs2981778** | * | * | 0.99/0.98 | 0.99/0.97 | 0.65/0.19 | 1.00/0.02 | 0.16/0.01 |
| **rs11523871** | * | * | * | 0.99/0.97 | 0.64/0.18 | 1.00/0.02 | 0.15/<0.01 |
| **rs3013236** | * | * | * | * | 0.64/0.18 | 1.00/0.02 | 0.18/0.01 |
| **rs2981804** | * | * | * | * | * | 0.51/<0.01 | 0.03/<0.01 |
| **rs2277244** | * | * | * | * | * | * | 0.63/0.02 |
| **rs1052715** | * | * | * | * | * | * | * |

**Table S6. Linkage disequilibrium (LD) matrix for *DMBT1* SNPs in CD and UC patients and controls.** Values are given as D’/r².
