# Supplementary material for: Intestinal DMBT1 Expression Is Modulated by Crohn’s Disease-Associated IL23R Variants and by a DMBT1 Variant Which Influences Binding of the Transcription Factors CREB1 and ATF-2
Source: PLoS One. 2013 Nov 5;8(11):e77773. doi: 10.1371/journal.pone.0077773 (PMC3818382; doi:10.1371/journal.pone.0077773)
Supplement: Table S7 — Association results of DMBT1 gene variants with UC. Risk allele frequencies (RAF), allelic test P-values (1 degree of freedom), and odds ratios (OR, shown for the riskallele) with 95% confidence intervals (CI) are depicted for the UC case-control panel. P-values <0.05 are highlighted in bold and P-values robust to multiple testing (P<0.0036) are highlighted in bold italic. P-values are based on 10,000,000 permutations. (DOC) [file pone.0077773.s011.doc]

| **SNP** | **Risk allele** | **Ulcerative colitis**  n=283 | | | **Controls**  n=972 |
| --- | --- | --- | --- | --- | --- |
|  |  | **RAF** | **empirical *P*-value** | **OR [95 % CI]** | **RAF** |
| rs2981745 | T | 0.423 | ***2.5 × 10^−4^*** | 1.50 [1.24-1.82] | 0.329 |
| rs2981778 | G | 0.687 | 0.535 | 1.09 [0.90-1.33] | 0.669 |
| rs11523871=  p.Pro42Thr | A | 0.675 | 0.958 | 1.02 [0.83-1.25] | 0.671 |
| rs3013236=  p.Leu54Ser | T | 0.693 | 0.478 | 1.11 [0.90-1.35] | 0.670 |
| rs2981804 | A | 0.541 | ***2.5 × 10^−3^*** | 1.31[1.08-1.58] | 0.475 |
| rs2277244=  p.His585Tyr | C | 0.962 | 0.372 | 0.90 [0.54-1.51] | 0.966 |
| rs1052715=  p.Pro1707Pro | A | 0.589 | 0.492 | 1.18 [0.98-1.43] | 0.547 |

**Table S7.** **Association results of *DMBT1* gene variants with UC.** Risk allele frequencies (RAF), allelic test *P*-values (1 degree of freedom), and odds ratios (OR, shown for the risk allele) with 95% confidence intervals (CI) are depicted for the UC case-control panel. *P*-values <0.05 are highlighted in **bold** and *P*-values robust to multiple testing (P<0.0036) are highlighted in ***bold italic***. P-values are based on 10,000,000 permutations.
