# Supplementary material for: Intestinal DMBT1 Expression Is Modulated by Crohn’s Disease-Associated IL23R Variants and by a DMBT1 Variant Which Influences Binding of the Transcription Factors CREB1 and ATF-2
Source: PLoS One. 2013 Nov 5;8(11):e77773. doi: 10.1371/journal.pone.0077773 (PMC3818382; doi:10.1371/journal.pone.0077773)
Supplement: Table S8 — Association between DMBT1 rs2981745 genotypes and CD disease characteristics in the subcohort of the Munich IBD center (n = 628) for which detailed phenotypic data based on the Montreal classification were available. For each variable, the number of patients included is given. PT, P-value for testing for differences between carriers and non-carriers of the T allele. ORT: corresponding odds ratios and 95% confidence intervals (95% CI). For age at diagnosis, P-values are given based on a median split. Significant P-values are depicted in bold. However, after Bonferroni correction for multiple testing, significance was lost. 1Disease behaviour was defined according to the Montreal classification. A stricturing disease phenotype was defined as presence of stenosis without penetrating disease. The diagnosis of stenosis was made surgically, endoscopically, or radiologically (using MRI enteroclysis). 2Immunosuppressive agents included azathioprine, 6-mercaptopurine, 6-thioguanin, methotrexate, infliximab and/or adalimumab. 3Only surgery related to CD-specific problems (e.g. fistulectomy, colectomy, ileostomy) was included. (DOC) [file pone.0077773.s012.doc]

| ***DMBT1***  **rs2981745** | **(1)**  **n=115** | **(2)**  **n=276** | **(3)**  **n=237** | **PT** | **ORT**  **[95% CI]** |
| --- | --- | --- | --- | --- | --- |
| **genotype** | **TT** | **CT** | **CC** |  |  |
| **Age at**  **diagnosis**  (yr)  *(n=600)*  Mean ± SD  Range | 29.88 ± 13.95  6 – 78 | 27.83 ± 12.10  7 – 71 | 27.35 ± 11.47  9 – 71 | 0.206 | 1.25  [0.88-1.77] |
| **Age at diagnosis**  *(n= 600)* | **n=105** | **n=255** | **n=240** |  |  |
| <=16 years  (A1) | 9  (8.60%) | 25  (9.80%) | 37  (15.42%) | **0.029** | 0.57  [0.35-0.94] |
| 17-40 years  (A2) | 76  (72.40%) | 195  (76.47%) | 175  (72.92%) | 0.567 | 1.13  [0.78-1.64] |
| > 40 years  (A3) | 20  (19.00%) | 35  (13.73%) | 28  (11.66%) | 0.229 | 1.36  [0.83-2.22] |
| **Location**  (*n= 596)* | **n=106** | **n=263** | **n=227** |  |  |
| Terminal ileum  (L1) | 18  (16.98%) | 41  (15.59%) | 29  (12.78%) | 0.342 | 1.30  [0.80-2.10] |
| Colon  (L2) | 17  (16.04%) | 38  (14.45%) | 21  (9.25%) | 0.057 | 1.72  [1.01-2.93] |
| Ileocolon  (L3) | 69  (65.09%) | 180  (68.44%) | 173  (76.21%) | **0.026** | 0.65  [0.44-0.95] |
| Upper GI  (L4) | 2  (1.89%) | 4  (1.52%) | 4  (1.76%) | 1.00 | 0.92  [0.26-3.30] |
| **Behaviour** ^1^  *(n= 520)* | **n=84** | **n=224** | **n=212** |  |  |
| Non-  stricturing -  Non-penetrating  (B1) | 27  (32.14%) | 63  (28.12%) | 41  (19.34%) | **0.013** | 1.72  [1.13-2.62] |
| Stricturing  (B2) | 27  (32.14%) | 54  (24.11%) | 64  (30.19%) | 0.371 | 0.83  [0.55-1.23] |
| Penetrating  (B3) | 30  (35.72%) | 107  (47.77%) | 107  (50.47%) | 0.181 | 0.79  [0.54-1.14] |
| **Use of**  **immuno-**  **suppressive**  **agents** ^2^  *(n= 482)* | 88  (76.5%) | 208  (75.3%) | 186  (78.5%) | 0.448 | 0.85  [0.58-1.25] |
| **Surgery**  **because of**  **CD** ^3^  *(n= 348)* | 57  (49.6%) | 155  (56.2%) | 136  (57.4%) | 0.457 | 0.87  [0.64-1.21] |
| **Fistulas**  *(n= 301)* | 46  (40.0%) | 133  (48.2%) | 122  (51.5%) | 0.097 | 0.79  [0.58-1.10] |
| **Stenosis**  *(n= 380)* | 67  (58.3%) | 162  (58.7%) | 151  (63.7%) | 0.208 | 0.81  [0.58-1.12] |

**Table S8. Association between *DMBT1* rs2981745 genotypes and CD disease characteristics in the subcohort of the Munich IBD center (n=628) for which detailed phenotypic data based on the Montreal classification were available.** For each variable, the number of patients included is given. PT, *P*-value for testing for differences between carriers and non-carriers of the T allele. ORT: corresponding odds ratios and 95% confidence intervals (95% CI). For age at diagnosis, *P*-values are given based on a median split. Significant *P*-values are depicted in bold. However, after Bonferroni correction for multiple testing, significance was lost.

1 Disease behaviour was defined according to the Montreal classification. A stricturing disease phenotype was defined as presence of stenosis without penetrating disease. The diagnosis of stenosis was made surgically, endoscopically, or radiologically (using MRI enteroclysis).

2 Immunosuppressive agents included azathioprine, 6-mercaptopurine, 6-thioguanin, methotrexate, and/or infliximab or adalimumab.

3 Only surgery related to CD-specific problems (e.g. fistulectomy, colectomy, ileostomy) was included.
