# Supplementary material for: Intestinal DMBT1 Expression Is Modulated by Crohn’s Disease-Associated IL23R Variants and by a DMBT1 Variant Which Influences Binding of the Transcription Factors CREB1 and ATF-2
Source: PLoS One. 2013 Nov 5;8(11):e77773. doi: 10.1371/journal.pone.0077773 (PMC3818382; doi:10.1371/journal.pone.0077773)
Supplement: Table S9 — Association between DMBT1 rs2981745 genotypes and UC disease characteristics in the subcohort of the Munich IBD center (n = 283) for which detailed phenotypic data based on the Montreal classification were available. For each variable, the number of patients included is given. PT, P-value for testing for differences between carriers and non-carriers of the T allele. ORT: corresponding odds ratios and 95% confidence intervals (95% CI). For age at diagnosis, age and BMI P-values are given based on a median split. Significant association is highlighted in bold. However, after Bonferroni correction for multiple testing, this significance was lost. (DOC) [file pone.0077773.s013.doc]

| ***DMBT1***  **rs2981745** | **(1)**  **n=49** | **(2)**  **n=137** | **(3)**  **n=97** | **PT** | **ORT**  **[95% CI]** |
| --- | --- | --- | --- | --- | --- |
| **genotype** | **TT** | **CT** | **CC** |  |  |
| Gender*(n=283)* |  |  |  |  |  |
| Male | 30  (61.20%) | 73  (53.28%) | 47  (48.45%) | 0.316 | 1.32  [0.81-2.15] |
| Female | 19  (38.80%) | 64  (46.72%) | 50  (51.55%) |  | 0.76  [0.46-1.24] |
| **Age (yrs)**  *(n=283)*  Mean ± SD  Range | 39.67± 11.43  21 – 68 | 43.34 ± 15.15  18 – 82 | 49.07 ± 14.88  24 – 89 | **0.001** | 0.44  [0.25-0.74] |
| **Age at diagnosis (yrs)**  *(n=267)* |  |  |  |  |  |
| Mean ± SD | 28.32±10.57 | 29.95±13.08 | 34.79±15.30 | **0.004** | 0.46  [0.26-0.79] |
| Range | 12 – 58 | 4 – 73 | 9 – 81 |  |  |
| Age at diagnosis*(n=267)* | **n=47** | **n=130** | **n=90** |  |  |
| ≤16 years (A1) | 4  (8.51%) | 13  (10.00%) | 7  (7.78%) | 0.821 | 1.26  [0.50-3.16] |
| 17-40 years (A2) | 37  (78.72%) | 95  (73.08%) | 59  (65.56%) | 0.151 | 1.54  [0.89-2.67] |
| > 40 years (A3) | 6  (12.77%) | 22  (16.92%) | 24  (26.66%) | **0.049** | 0.52  [0.28-0.96) |
| **BMI (kg/m²)**  *(n=209)* |  |  |  |  |  |
| Mean ± SD | 24.29±6.83 | 23.45±3.73 | 24.31±4.15 | 0.473 | 0.81  [0.44-1.48] |
| Range | 15-54 | 15-37 | 16-41 |  |  |
| Location*(n=260)* | **n=44** | **n=128** | **n=88** |  |  |
| Proctitis (E1) | 4  (9.09%) | 12  (9.38%) | 8  (9.09%) | 0.829 | 1.02  [0.42-2.50] |
| Left-sided UC (E2) | 11  (25.00%) | 44  (34.38%) | 41  (46.59%) | **0.030** | 0.54  [0.32-0.91] |
| Extensive UC (E3) | 29  (65.91%) | 72  (56.25%) | 39  (44.32%) | **0.035** | 1.79  [1.06-3.00] |
| **Extra-intestinal manifestations**  *(n=55)* | 14  (28.6%) | 23  (16.8%) | 18  (18.6%) | 0.872 | 1.09  [0.58-2.04] |
| **Use of immunosuppressive agents** *(n=202)* | 38  (77.6%) | 95  (69.3%) | 69  (71.1%) | 0.889 | 1.02  [0.59-1.75] |
| **Abscesses**  *(n=12)* | 3  (6.1%) | 5  (3.6%) | 4  (4.1%) | 1.00 | 1.04  [0.31-3.56] |

**Table S9. Association between *DMBT1* rs2981745 genotypes and UC disease characteristics in the subcohort of the Munich IBD center (n=283) for which detailed phenotypic data based on the Montreal classification were available.** For each variable, the number of patients included is given. PT, *P*-value for testing for differences between carriers and non-carriers of the T allele. ORT: corresponding odds ratios and 95% confidence intervals (95% CI). For age at diagnosis, age and BMI *P*-values are given based on a median split. Significant association is highlighted in bold. However, after Bonferroni correction for multiple testing, this significance was lost.
