# Supplementary material for: Intestinal DMBT1 Expression Is Modulated by Crohn’s Disease-Associated IL23R Variants and by a DMBT1 Variant Which Influences Binding of the Transcription Factors CREB1 and ATF-2
Source: PLoS One. 2013 Nov 5;8(11):e77773. doi: 10.1371/journal.pone.0077773 (PMC3818382; doi:10.1371/journal.pone.0077773)
Supplement: Table S10 — Association between DMBT1 rs2981804 genotypes and CD disease characteristics in the subcohort of the Munich IBD center (n = 626) for which detailed phenotypic data based on the Montreal classification were available. For each variable, the number of patients included is given. PG, P-value for testing for differences between carriers and non-carriers of the G allele. ORG: corresponding odds ratios and 95% confidence intervals (95% CI). For age at diagnosis P-values are given based on a median split. 1Disease behaviour was defined according to the Montreal classification. A stricturing disease phenotype was defined as presence of stenosis without penetrating disease. The diagnosis of stenosis was made surgically, endoscopically, or radiologically (using MRI enteroclysis). 2Immunosuppressive agents included azathioprine, 6-mercaptopurine, 6-thioguanin, methotrexate, infliximab and/or adalimumab. 3Only surgery related to CD-specific problems (e.g. fistulectomy, colectomy, ileostomy) was included. (DOC) [file pone.0077773.s014.doc]

| ***DMBT1***  **rs2981804** | **(1)**  **n=197** | **(2)**  **n=307** | **(3)**  **n=122** | **PA** | **ORA**  **[95% CI]** |
| --- | --- | --- | --- | --- | --- |
| **genotype** | **AA** | **GA** | **GG** |  |  |
| **Age at**  **diagnosis**  (yr)  *(n=598)*  Mean ± SD  Range | 27.56 ± 11.91  11 – 73 | 28.09 ± 12.33  7 – 78 | 28.46 ± 12.61  6 – 71 | 0.659 | 0.91  [0.64-1.32] |
| **Age at diagnosis**  *(n= 598)* | **n=190** | **n=291** | **n=117** |  |  |
| <=16 years  (A1) | 19  (10.00%) | 38  (13.06%) | 14  (11.97%) | 1.000 | 0.98  [0.53-1.84] |
| 17-40 years  (A2) | 149  (78.42%) | 212  (72.85%) | 83  (70.94%) | 0.409 | 1.23  [0.79-1.93] |
| > 40 years  (A3) | 22  (11.58%) | 41  (14.09%) | 20  (17.09%) | 0.296 | 0.73  [0.42-1.27] |
| **Location**  (*n= 594)* | **n=188** | **n=290** | **n=116** |  |  |
| Terminal ileum  (L1) | 26  (13.83%) | 41  (14.14%) | 21  (18.10%) | 0.307 | 0.73  [0.43-1.26] |
| Colon  (L2) | 21  (11.17%) | 37  (12.76%) | 18  (15.52%) | 0.356 | 0.75  [0.42-1.33] |
| Ileocolon  (L3) | 137  (72.87%) | 208  (71.72%) | 75  (64.66%) | 0.113 | 1.42  [0.92-2.18] |
| Upper GI  (L4) | 4  (2.13%) | 4  (1.38%) | 2  (1.72%) | 1.000 | 0.97  [0.20-4.63] |
| **Behaviour**^1^  *(n= 519)* | **n=167** | **n=249** | **n=103** |  |  |
| Non-stricturing -Non-penetrating  (B1) | 41  (24.55%) | 65  (26.21%) | 25  (24.27%) | 0.899 | 1.07  [0.65-1.76] |
| Stricturing  (B2) | 44  (26.35%) | 75  (29.84%) | 25  (24.27%) | 0.461 | 1.25  [0.76-2.06] |
| Penetrating  (B3) | 82  (49.10%) | 109  (43.95%) | 53  (51.46%) | 0.323 | 0.80  [0.52-1.23] |
| **Use of**  **immuno-suppressive agents** ^2^  *(n= 480)* | 152  (77.2%) | 235  (76.5%) | 93  (76.2%) | 0.905 | 1.03  [0.65-1.65] |
| **Surgery**  **because of CD** ^3^  *(n= 347)* | 113  (57.4%) | 172  (56.0%) | 62  (50.8%) | 0.265 | 1.26  [0.85-1.87] |
| **Fistulas**  *(n= 301)* | 102  (51.8%) | 138  (45.0%) | 61  (50.0%) | 0.687 | 0.90  [0.61-1.35] |
| **Stenosis**  *(n= 378)* | 121  (61.4%) | 179  (58.3%) | 78  (63.9%) | 0.410 | 0.83  [0.55-1.25] |

**Table S10. Association between *DMBT1* rs2981804 genotypes and CD disease characteristics in the subcohort of the Munich IBD center (n=626) for which detailed phenotypic data based on the Montreal classification were available.** For each variable, the number of patients included is given. PG, *P*-value for testing for differences between carriers and non-carriers of the G allele. ORG: corresponding odds ratios and 95% confidence intervals (95% CI). For age at diagnosis *P*-values are given based on a median split.

^1^ Disease behaviour was defined according to the Montreal classification. A stricturing disease phenotype was defined as presence of stenosis without penetrating disease. The diagnosis of stenosis was made surgically, endoscopically, or radiologically (using MRI enteroclysis).

^2^ Immunosuppressive agents included azathioprine, 6-mercaptopurine, 6-thioguanin, methotrexate, and/or infliximab or adalimumab.

^3^ Only surgery related to CD-specific problems (e.g. fistulectomy, colectomy, ileostomy) was included.
