# Supplementary material for: Intestinal DMBT1 Expression Is Modulated by Crohn’s Disease-Associated IL23R Variants and by a DMBT1 Variant Which Influences Binding of the Transcription Factors CREB1 and ATF-2
Source: PLoS One. 2013 Nov 5;8(11):e77773. doi: 10.1371/journal.pone.0077773 (PMC3818382; doi:10.1371/journal.pone.0077773)
Supplement: Table S11 — DMBT1 gene markers in CD – Haplotype frequencies (HF), P -values, and odds ratios (OR) with 95% confidence intervals (CI). P-values for individual haplotypes are presented for all haplotypes with a frequency of at least 1% in the whole sample and with an omnibus haplotype P-value <0.05. Significant P-values (<0.05) are highlighted in bold and significant P-values robust to multiple testing (P<2.5×10^−3^ for omnibus P-values, P<4.8×10^−4^ for detailed haplotype P-values) are highlighted in bold italic. (DOC) [file pone.0077773.s015.doc]

| **Haplotype** | **Crohn’s disease** | | | **Controls** |
| --- | --- | --- | --- | --- |
|  | **HF** | ***P-*value** | **OR [95 % CI]** | **HF** |
| rs2981745-rs2981778 |  | ***1.28x10^-10^*** |  |  |
| TA | 0.27 | **1.47x10^-3^** | 0.79 [0.68-0.91] | 0.32 |
| CA | 0.03 | **8.07x10^-3^** | 1.92 [1.19-3.11] | 0.01 |
| TG | 0.10 | **3*.01x10^-10^*** | 5.36 [3.18-9.04] | <0.01 |
| CG | 0.60 | **5.47x10^-4^** | 0.79 [0.69-0.90] | 0.66 |
| rs2981778-rs11523871 |  | ***7.45x10^-5^*** |  |  |
| AC | 0.28 | **5.49x10^-3^** | 0.82 [0.71-0.94] | 0.33 |
| GC | 0.01 | **1.89x10^-3^** | 6.76 [2.02-22.57] | <0.01 |
| AA | 0.01 | **8.40x10^-3^** | 3.02 [1.33-6.87] | <0.01 |
| GA | 0.69 | 1.37x10^-1^ | 1.11 [0.97-1.27] | 0.67 |
| rs11523871-rs3013236 |  | ***4.63x10^-4^*** |  |  |
| CC | 0.28 | **3.15x10^-3^** | 0.80 [0.70-0.93] | 0.32 |
| CT | 0.02 | **5.11x10^-4^** | 3.71 [1.77-7.77] | <0.01 |
| AT | 0.69 | 1.60x10^-1^ | 1.11 [0.96-1.28] | 0.67 |
| rs3013236-rs2981804 |  | ***1.58x10^-14^*** |  |  |
| CG | 0.29 | 9.80x10^-1^ | 1.00 [0.85-1.17] | 0.27 |
| TG | 0.15 | ***2.33x10^-8^*** | 0.65 [0.56-0.76] | 0.25 |
| CA | <0.01 | **2.15x10^-2^** | <0.01 [<0.01-0.29] | 0.06 |
| TA | 0.56 | ***9.87x10^-13^*** | 1.62 [1.42-1.85] | 0.42 |
| rs2981804-rs2277244 |  | ***8.37x10^-7^*** |  |  |
| GT | 0.02 | 5.05x10^-2^ | 0.61 [0.37-1.01] | 0.03 |
| GC | 0.42 | ***2.64x10^-6^*** | 0.73 [0.64-0.83] | 0.50 |
| AC | 0.56 | ***1.68x10^-7^*** | 1.42 [1.25-1.62] | 0.47 |
| rs2981745-rs2981778-rs11523871 |  | ***1.41x10^-8^*** |  |  |
| TAC | 0.27 | **4.92x10^-4^** | 0.77 [0.66-0.89] | 0.32 |
| CAC | 0.02 | **1.90x10^-2^** | 1.97 [1.18-3.47] | <0.01 |
| TGC | 0.01 | **2.24x10^-3^** | 6.19 [1.92-19.92] | <0.01 |
| TGA | 0.09 | ***1.87x10^-8^*** | 8.35 [3.98-17.50] | <0.01 |
| CGA | 0.60 | **4.05x10^-4^** | 0.79 [0.69-0.90] | 0.66 |
| rs2981778-rs11523871-rs3013236 |  | ***2.04x10^-3^*** |  |  |
| ACC | 0.28 | **4.42x10^-3^** | 0.81 [0.70-0.94] | 0.32 |
| GCT | 0.01 | **2.67x10^-3^** | 23.10 [2.98-179.27] | <0.01 |
| GAT | 0.69 | 1.35x10^-1^ | 1.11 [0.97-1.27] | 0.67 |
| rs11523871-rs3013236-rs2981804 |  | ***3.02x10^-16^*** |  |  |
| CCG | 0.28 | 5.00x10^-1^ | 0.95 [0.82-1.10] | 0.27 |
| ATG | 0.13 | ***1.75x10^-9^*** | 0.62 [0.54-0.73] | 0.25 |
| CCA | <0.01 | **1.18x10^-2^** | <0.01 [<0.01-0.13] | 0.05 |
| ATA | 0.56 | ***2.46x10^-11^*** | 1.57 [1.38-1.79] | 0.42 |
| rs3013236-rs2981804 -rs2277244 |  | ***6.61x10^-12^*** |  |  |
| TGT | 0.01 | 1.22x10^-1^ | 0.67 [0.40-1.11] | 0.02 |
| CGC | 0.29 | 9.68x10^-1^ | 1.00 [0.86-1.16] | 0.27 |
| TGC | 0.13 | ***8.38x10^-8^*** | 0.65 [0.56-0.76] | 0.23 |
| CAC | <0.01 | **2.25x10^-2^** | <0.01[<0.01-0.39] | 0.06 |
| TAC | 0.56 | ***2.30x10^-13^*** | 1.64 [1.44-1.87] | 0.41 |
| rs2981804 -rs2277244-rs1052715 |  | ***1.16x10^-13^*** |  |  |
| GTG | 0.01 | 1.48x10^-1^ | 0.64 [0.35-1.17] | 0.02 |
| GCG | 0.24 | 6.53x10^-1^ | 0.96 [0.81-1.14] | 0.21 |
| ACG | 0.17 | 1.94x10^-1^ | 0.89 [0.75-1.06] | 0.22 |
| GCA | 0.18 | ***7.80x10^-8^*** | 0.63 [0.53-0.74] | 0.29 |
| ACA | 0.39 | ***7.60x10^-12^*** | 1.68 [1.45-1.95] | 0.25 |
| rs2981745-rs2981778-rs11523871-rs3013236 |  | ***7.35x10^-6^*** |  |  |
| TACC | 0.26 | **2.39x10^-4^** | 0.76 [0.66-0.88] | 0.32 |
| CACC | 0.02 | **1.47x10^-2^** | 2.07 [1.15-3.71] | <0.01 |
| TGAT | 0.09 | ***1.53x10^-6^*** | 13.40 [4.65-38.61] | <0.01 |
| CGAT | 0.60 | **3.74x10^-4^** | 0.79 [0.69-0.90] | 0.66 |
| rs2981778-rs11523871-rs3013236-rs2981804 |  | ***4.39x10^-16^*** |  |  |
| ACCG | 0.28 | 4.62x10^-1^ | 0.95 [0.82-1.10] | 0.27 |
| GATG | 0.13 | ***1.93x10^-9^*** | 0.63 [0.54-0.73] | 0.25 |
| ACCA | <0.01 | **9.26x10^-3^** | <0.01[<0.01-0.08] | 0.05 |
| GATA | 0.56 | ***1.15x10^-11^*** | 1.58 [1.38-1.80] | 0.42 |
| rs11523871-rs3013236-rs2981804-rs2277244 |  | ***1.92x10^-13^*** |  |  |
| ATGT | 0.02 | 1.23x10^-1^ | 0.67 [0.40-1.11] | 0.02 |
| CCGC | 0.28 | 4.82x10^-1^ | 0.95 [0.82-1.10] | 0.27 |
| ATGC | 0.12 | ***6.93x10^-9^*** | 0.62 [0.53-0.73] | 0.23 |
| ATAC | 0.55 | ***6.80x10^-12^*** | 1.59 [1.39-1.82] | 0.41 |
| rs3013236-rs2981804-rs2277244-rs1052715 |  | ***3.60x10^-13^*** |  |  |
| TGTG | 0.01 | 2.88x10^-1^ | 0.72 [0.39-1.32] | 0.02 |
| CGCG | 0.13 | 4.67x10^-1^ | 1.09 [0.86-1.37] | 0.09 |
| TGCG | 0.11 | 3.00x10^-1^ | 0.89 [0.72-1.11] | 0.11 |
| TACG | 0.17 | 9.94x10^-1^ | 1.00 [0.99-1.01] | 0.20 |
| CGCA | 0.16 | 5.52x10^-1^ | 0.94 [0.78-1.14] | 0.18 |
| TGCA | 0.02 | ***1.55x10^-11^*** | 0.33 [0.23-0.45] | 0.12 |
| CACA | <0.01 | 7.27x10^-2^ | <0.01 [<0.01-2.05] | 0.03 |
| TACA | 0.39 | ***1.95x10^-16^*** | 1.90 [1.63-2.21] | 0.21 |
| rs2981745-rs2981778-rs11523871-rs3013236-rs2981804 |  | ***1.38x10^-15^*** |  |  |
| TACCG | 0.26 | 1.33x10^-1^ | 0.89 [0.76-1.04] | 0.27 |
| CACCG | 0.02 | **1.56x10^-2^** | 2.06 [1.15-3.70] | <0.01 |
| TGATG | 0.02 | **2.40x10^-3^** | 74.70 [18.05-309.22] | <0.01 |
| CGATG | 0.12 | ***1.89x10^-12^*** | 0.57 [0.48-0.66] | 0.25 |
| TACCA | <0.01 | **2.59x10^-2^** | <0.01[<0.01-0.39] | 0.05 |
| TGATA | 0.07 | ***6.71x10^-6^*** | 32.2 [7.11-145.81] | <0.01 |
| CGATA | 0.48 | **4.01x10^-3^** | 1.21 [1.06-1.38] | 0.42 |
| rs2981778-rs11523871-rs3013236-rs2981804-rs2277244 |  | **4.52x10^-13^** |  |  |
| GATGT | 0.02 | 1.26x10^-1^ | 0.67 [0.40-1.12] | 0.02 |
| ACCGC | 0.28 | 4.85x10^-1^ | 0.95 [0.82-1.10] | 0.27 |
| GATGC | 0.12 | ***5.65x10^-9^*** | 0.62 [0.53-0.73] | 0.23 |
| ACCAC | <0.01 | **2.36x10^-2^** | <0.01 [<0.01-0.39] | 0.05 |
| GATAC | 0.55 | ***3.78x10^-12^*** | 1.60 [1.40-1.83] | 0.41 |
| rs11523871-rs3013236-rs2981804-rs2277244-rs1052715 |  | ***8.92x10^-18^*** |  |  |
| ATGTG | 0.01 | 2.84x10^-1^ | 0.72 [0.39-1.32] | 0.02 |
| CCGCG | 0.12 | 9.09x10^-1^ | 1.01 [0.85-1.20] | 0.09 |
| ATGCG | 0.10 | 1.05x10^-1^ | 0.83 [0.66-1.04] | 0.11 |
| CCACG | <0.01 | **4.58x10^-2^** | <0.01[<0.01-0.87] | 0.03 |
| ATACG | 0.16 | 7.04x10^-1^ | 0.97 [0.80-1.16] | 0.20 |
| CCGCA | 0.16 | 3.14x10^-1^ | 0.91 [0.75-1.10] | 0.18 |
| ATGCA | 0.02 | ***9.87x10^-12^*** | 0.32 [0.23-0.44] | 0.12 |
| CCACA | <0.01 | 9.45x10^-2^ | <0.01 [<0.01-3.18] | 0.03 |
| ATACA | 0.38 | ***9.26x10^-16^*** | 1.87 [1.61-2.18] | 0.21 |
| rs2981745-rs2981778-rs11523871-rs3013236-rs2981804-rs2277244 |  | ***1.17x10^-13^*** |  |  |
| CGATGT | 0.01 | 5.29x10^-2^ | 0.60 [0.35-1.00] | 0.02 |
| TACCGC | 0.26 | 1.29x10^-1^ | 0.89 [0.76-1.04] | 0.27 |
| CACCGC | 0.02 | **1.52x10^-2^** | 2.07 [1.15-3.73] | <0.01 |
| CGATGC | 0.10 | ***1.32x10^-11^*** | 0.53 [0.48-0.66] | 0.22 |
| TACCAC | <0.01 | **4.60x10^-2^** | <0.01 [<0.01-0.91] | 0.05 |
| TGATAC | 0.07 | ***1.02x10^-5^*** | 37.90 [7.55-190.25] | <0.01 |
| CGATAC | 0.48 | **2.09x10^-3^** | 1.22 [1.07-1.38] | 0.41 |
| rs2981778-rs11523871-rs3013236-rs2981804-rs2277244-rs1052715 |  | ***1.80x10^-17^*** |  |  |
| GATGTG | 0.01 | 2.90x10^-1^ | 0.72 [0.39-1.32] | 0.02 |
| ACCGCG | 0.12 | 9.18x10^-1^ | 1.01 [0.84-1.22] | 0.09 |
| GATGCG | 0.10 | 1.08x10^-1^ | 0.83 [0.67-1.04] | 0.11 |
| GATACG | 0.16 | 6.83x10^-1^ | 0.96 [0.80-1.15] | 0.20 |
| ACCGCA | 0.16 | 3.20x10^-1^ | 0.91 [0.75-1.10] | 0.18 |
| GATGCA | 0.02 | ***8.32x10^-12^*** | 0.32 [0.23-0.44] | 0.12 |
| ACCACA | <0.01 | 1.23x10^-1^ | <0.01 [<0.01-6.77] | 0.03 |
| GATACA | 0.38 | ***3.32x10^-16^*** | 1.89 [1.62-2.20] | 0.21 |
| rs2981745-rs2981778-rs11523871-rs3013236-rs2981804-rs2277244-rs1052715 |  | ***6.14x10^-18^*** |  |  |
| CGATGTG | 0.01 | 9.88x10^-2^ | 0.62 [0.33-1.14] | 0.02 |
| CGATACG | 0.13 | **7.33x10^-3^** | 0.77 [0.64-0.93] | 0.23 |
| CGATGCG | 0.09 | **2.81x10^-2^** | 0.78 [0.63-0.97] | 0.10 |
| TACCGCA | 0.15 | 1.17x10^-1^ | 0.85 [0.70-1.04] | 0.20 |
| TACCACA | <0.01 | 6.85x10^-2^ | <0.01 [<0.01-1.50] | 0.03 |
| TACCGCG | 0.11 | 6.57x10^-1^ | 0.95 [0.74-1.21] | 0.06 |
| CGATGCA | 0.02 | ***4.04x10^-13^*** | 0.25 [0.17-0.36] | 0.13 |
| CGATACA | 0.34 | ***1.53x10^-8^*** | 1.54 [1.33-1.79] | 0.17 |
| TGATACG | 0.03 | ***2.92x10^-5^*** | 29.40 [6.03-143.3] | <0.01 |

**Table S11. *DMBT1* gene markers in CD – Haplotype frequencies (HF), *P* -values, and odds ratios (OR) with 95% confidence intervals (CI).** *P-*values for individual haplotypes are presented for all haplotypes with a frequency of at least 1% in the whole sample and with an omnibus haplotype *P-*value < 0.05. Significant *P-*values (<0.05) are highlighted in bold and significant *P*-values robust to multiple testing (*P*<2.5x10^-3^ *for omnibus P*-values, *P*<4.8x10^-4^ for detailed haplotype *P-*values) are highlighted in *bold italic*.
