# Supplementary material for: Intestinal DMBT1 Expression Is Modulated by Crohn’s Disease-Associated IL23R Variants and by a DMBT1 Variant Which Influences Binding of the Transcription Factors CREB1 and ATF-2
Source: PLoS One. 2013 Nov 5;8(11):e77773. doi: 10.1371/journal.pone.0077773 (PMC3818382; doi:10.1371/journal.pone.0077773)
Supplement: Table S12 — DMBT1 gene markers in UC – Haplotype frequencies (HF), P -values, and odds ratios (OR) with 95% confidence intervals (CI). P-values for individual haplotypes are presented for all haplotypes with a frequency of at least 1% in the whole sample and with an omnibus haplotype P-value <0.05. Significant P-values (<0.05) are highlighted in bold and significant P-values robust to multiple testing (P<2.5×10^−3^ for omnibus P-values, P<4.810−4 for detailed haplotype P-values) are highlighted in bold italic. (DOC) [file pone.0077773.s016.doc]

| **Haplotype** | **Ulcerative colitis** | | | **Controls** |
| --- | --- | --- | --- | --- |
|  | **HF** | ***P*-value** | **OR [95 % CI]** | **HF** |
| rs2981745-rs2981778 |  | ***1.37x10^-11^*** |  |  |
| TA | 0.28 | 9.56x10^-2^ | 0.84 [0.68-1.03] | 0.32 |
| CA | 0.03 | **7.31x10^-3^** | 2.44 [1.27-4.68] | 0.01 |
| TG | 0.13 | ***2.39x10^-11^*** | 6.23 [3.64-10.66] | <0.01 |
| CG | 0.55 | ***1.11x10^-5^*** | 0.65 [0.54-0.79] | 0.66 |
| rs2981778-rs11523871 |  | **5.35x10^-3^** |  |  |
| AC | 0.30 | 2.92x10^-1^ | 0.90 [0.74-1.09] | 0.33 |
| GC | 0.02 | **9.52x10^-4^** | 8.33 [2.37-29.32] | <0.01 |
| GA | 0.67 | 9.91x10^-1^ | 1.00 [0.99-1.01] | 0.67 |
| rs11523871-rs3013236 |  | ***1.26x10^-3^*** |  |  |
| CC | 0.30 | 2.53x10^-1^ | 0.89 [0.72-1.09] | 0.32 |
| CT | 0.03 | ***3.02x10^-4^*** | 4.43 [1.98-9.92] | <0.01 |
| AT | 0.67 | 9.67x10^-1^ | 1.00 [0.83-1.20] | 0.67 |
| rs3013236-rs2981804 |  | ***1.70x10^-4^*** |  |  |
| CG | 0.31 | 5.51x10^-1^ | 1.07 [0.86-1.34] | 0.27 |
| TG | 0.15 | **9.45x10^-4^** | 0.70 [0.57-0.87] | 0.25 |
| CA | <0.01 | **3.27x10^-2^** | 0.21 [0.04-0.88] | 0.06 |
| TA | 0.54 | ***3.66x10^-5^*** | 1.48 [1.23-1.78] | 0.42 |
| rs2981804-rs2277244 |  | **1.67x10^-2^** |  |  |
| GT | 0.03 | 9.94x10^-1^ | 1.00 [0.99-1.01] | 0.03 |
| GC | 0.43 | **4.37x10^-3^** | 0.76 [0.63-0.92] | 0.50 |
| AC | 0.53 | **5.75x10^-3^** | 1.30 [1.08-1.57] | 0.47 |
| rs2981745-rs2981778-rs11523871 |  | ***2.10x10^-11^*** |  |  |
| TAC | 0.27 | 5.39x10^-2^ | 0.82 [0.66-1.00] | 0.32 |
| CAC | 0.03 | **1.15x10^-3^** | 3.32 [1.61-6.84] | <0.01 |
| TGC | 0.02 | **2.08x10^-3^** | 7.87 [2.12-29.26] | <0.01 |
| TGA | 0.12 | ***4.93x10^-9^*** | 8.56 [4.17-17.58] | <0.01 |
| CGA | 0.55 | ***3.67x10^-6^*** | 0.64 [0.53-0.77] | 0.66 |
| rs2981778-rs11523871-rs3013236 |  | **5.07x10^-3^** |  |  |
| ACC | 0.30 | 2.98x10^-1^ | 0.90 [0.73-1.10] | 0.32 |
| GCT | 0.02 | **1.42x10^-3^** | 31.3 [3.78-259.04] | <0.01 |
| GAT | 0.67 | 9.93x10^-1^ | 1.00 [0.97-1.02] | 0.67 |
| rs11523871-rs3013236-rs2981804 |  | ***5.41x10^-6^*** |  |  |
| CCG | 0.30 | 7.18x10^-1^ | 1.04 [0.84-1.29] | 0.27 |
| ATG | 0.14 | ***3.21x10^-4^*** | 0.67 [0.54-0.84] | 0.25 |
| CCA | <0.01 | **3.25x10^-2^** | 0.21 [0.05-0.88] | 0.05 |
| CTA | 0.02 | **8.03x10^-4^** | 11.10 [2.71-45.45] | <0.01 |
| ATA | 0.52 | ***4.29x10^-4^*** | 1.40 [1.16-1.69] | 0.42 |
| rs3013236-rs2981804-rs2277244 |  | ***2.35x10^-4^*** |  |  |
| TGT | 0.02 | 8.06x10^-1^ | 1.08 [0.59-1.99] | 0.02 |
| CGC | 0.29 | 5.55x10^-1^ | 1.07 [0.85-1.34] | 0.27 |
| TGC | 0.13 | ***4.93x10^-4^*** | 0.67 [0.54-0.84] | 0.23 |
| CAC | <0.01 | **2.83x10^-2^** | 0.22 [0.55-0.85] | 0.06 |
| TAC | 0.56 | ***4.82x10^-5^*** | 1.47 [1.77-1.22] | 0.41 |
| rs2981804-rs2277244-rs1052715 |  | ***3.45x10^-7^*** |  |  |
| GTG | 0.01 | 7.08x10^-1^ | 1.16 [0.53-2.52] | 0.02 |
| GCG | 0.24 | 5.77x10^-1^ | 0.93 [0.73-1.19] | 0.21 |
| ACG | 0.17 | **1.72x10^-2^** | 0.72 [0.55-0.94] | 0.22 |
| GCA | 0.18 | **3.03x10^-3^** | 0.69 [0.54-0.88] | 0.29 |
| ACA | 0.39 | ***2.45x10^-7^*** | 1.71 [1.39-2.10] | 0.25 |
| rs2981745-rs2981778-rs11523871-rs3013236 |  | ***2.73x10^-7^*** |  |  |
| TACC | 0.27 | **4.36x10^-2^** | 0.81 [0.66-0.99] | 0.32 |
| CACC | 0.03 | **9.59x10^-4^** | 3.52 [1.67-7.43] | <0.01 |
| TGAT | 0.12 | ***5.57x10^-7^*** | 12.50 [4.65-33.58] | <0.01 |
| CGAT | 0.55 | ***3.40x10^-6^*** | 0.64 [0.53-0.77] | 0.66 |
| rs2981778-rs11523871-rs3013236-rs2981804 |  | ***2.34x10^-4^*** |  |  |
| ACCG | 0.30 | 7.40x10^-1^ | 1.04 [0.82-1.31] | 0.27 |
| GATG | 0.14 | ***3.62x10^-4^*** | 0.68 [0.55-0.84] | 0.25 |
| ACCA | <0.01 | **3.73x10^-2^** | 0.21 [0.05-0.91] | 0.05 |
| GATA | 0.52 | ***3.49x10^-4^*** | 1.40 [1.16-1.68] | 0.42 |
| rs11523871-rs3013236-rs2981804-rs2277244 |  | ***3.83x10^-6^*** |  |  |
| ATGT | 0.02 | 9.89x10^-1^ | 1.00 [0-99-1.01] | 0.02 |
| CCGC | 0.30 | 7.42x10^-1^ | 1.04 [0.83-1.31] | 0.27 |
| ATGC | 0.12 | ***1.90x10^-4^*** | 0.64 [0.51-0.81] | 0.23 |
| CCAC | <0.01 | **2.95x10^-2^** | 0.21 [0.05-0.86] | 0.05 |
| CTAC | 0.02 | **8.13x10^-4^** | 11.10 [2.71-45.45] | <0.01 |
| ATAC | 0.51 | ***4.55x10^-4^*** | 1.40 [1.16-1.69] | 0.41 |
| rs3013236-rs2981804-rs2277244-rs1052715 |  | ***6.20x10^-8^*** |  |  |
| TGTG | 0.02 | 5.15x10^-1^ | 1.29 [0.60-2.78] | 0.02 |
| CGCG | 0.12 | 9.04x10^-1^ | 1.02 [0.74-1.41] | 0.09 |
| TGCG | 0.12 | 5.89x10^-1^ | 0.92 [0.68-1.24] | 0.11 |
| CACG | <0.01 | 1.69x10^-1^ | 0.01 [<0.01-6.93] | 0.03 |
| TACG | 0.14 | 9.02x10^-2^ | 0.79 [0.60-1.04] | 0.20 |
| CGCA | 0.18 | 5.57x10^-1^ | 1.08 [0.84-1.40] | 0.19 |
| TGCA | <0.01 | ***1.46x10^-5^*** | 0.30 [0.17-0.52] | 0.12 |
| CACA | <0.01 | **5.93x10^-2^** | 0.19 [0.03-1.07] | 0.03 |
| TACA | 0.39 | ***7.24x10^-10^*** | 1.91 [1.56-2.35] | 0.21 |
| rs2981745-rs2981778-rs11523871-rs3013236-rs2981804 |  | ***3.53x10^-10^*** |  |  |
| TACCG | 0.27 | 5.57x10^-1^ | 0.94 [0.91-0.96] | 0.27 |
| CACCG | 0.03 | **1.31x10^-3^** | 3.41 [1.61-7.21] | <0.01 |
| TGATG | 0.03 | **5.01x10^-4^** | 61.10 [6.02-620.02] | <0.01 |
| CGATG | 0.11 | ***2.73x10^-6^*** | 0.57 [0.45-0.72] | 0.25 |
| TACCA | <0.01 | **3.58x10^-2^** | 0.21 [0.05-0.90] | 0.05 |
| TGATA | 0.09 | ***3.42x10^-6^*** | 32.60 [7.50-141.70] | <0.01 |
| CGATA | 0.44 | 8.65x10^-1^ | 1.02 [0.81-1.28] | 0.42 |
| rs2981778-rs11523871-rs3013236-rs2981804-rs2277244 |  | ***1.82x10^-5^*** |  |  |
| GATGT | 0.02 | 7.77x10^-1^ | 1.10 [0.57-2.13] | 0.02 |
| ACCGC | 0.30 | 7.13x10^-1^ | 1.04 [0.84-1.28] | 0.27 |
| GATGC | 0.12 | ***1.61x10^-4^*** | 0.64 [0.51-0.81] | 0.23 |
| ACCAC | <0.01 | **3.28x10^-2^** | 0.22 [0.06-0.88] | 0.05 |
| GATAC | 0.51 | **5.17x10^-4^** | 01.39 [1.15-1.67] | 0.41 |
| rs11523871-rs3013236-rs2981804-rs2277244-rs1052715 |  | ***6.96x10^-9^*** |  |  |
| ATGTG | 0.02 | 5.83x10^-1^ | 1.24 [0.58-2.67] | 0.02 |
| CCGCG | 0.12 | **3.06x10^-2^** | 1.03 [0.74-1.43] | 0.09 |
| ATGCG | 0.11 | 3.25x10^-1^ | 0.85 [0.62-1.17] | 0.11 |
| CCACG | <0.01 | 1.68x10^-1^ | 0.01 [<0.01-6.71] | 0.03 |
| ATACG | 0.13 | **4.65x10^-2^** | 0.76 [0.57-0.99] | 0.20 |
| CCGCA | 0.18 | 7.85x10^-1^ | 1.04 [0.78-1.38] | 0.18 |
| ATGCA | <0.01 | ***1.50x10^-5^*** | 0.30 [0.18-0.52] | 0.12 |
| CCACA | <0.01 | 6.19x10^-2^ | 0.23 [0.05-1.08] | 0.03 |
| ATACA | 0.38 | ***1.58x10^-8^*** | 1.81 [1.47-2.22] | 0.21 |
| rs2981745-rs2981778-rs11523871-rs3013236-rs2981804-rs2277244 |  | ***1.39x10^-10^*** |  |  |
| CGATGT | 0.02 | 8.70x10^-1^ | 0.95 [0.49-1.84] | 0.02 |
| TACCGC | 0.27 | 5.42x10^-1^ | 0.94 [0.75-1.16] | 0.27 |
| CACCGC | 0.03 | **1.30x10^-3^** | 3.44 [1.62-7.32] | <0.01 |
| CGATGC | 0.09 | ***1.89x10^-6^*** | 0.54 [0.42-0.69] | 0.22 |
| TACCAC | <0.01 | **3.19x10^-2^** | 0.22 [0.06-0.88] | 0.05 |
| TGATAC | 0.08 | ***3.65x10^-6^*** | 37.50 [8.07-174.15] | <0.01 |
| CGATAC | 0.43 | 8.79x10^-1^ | 1.01 [0.89-1.15] | 0.41 |
| rs2981778-rs11523871-rs3013236-rs2981804-rs2277244-rs1052715 |  | ***1.21x10^-8^*** |  |  |
| GATGTG | 0.02 | 5.05x10^-1^ | 1.29 [0.61-2.73] | 0.02 |
| ACCGCG | 0.12 | 8.84x10^-1^ | 1.03 [0.69-1.53] | 0.09 |
| GATGCG | 0.12 | 3.30x10^-1^ | 0.85 [0.62-1.17] | 0.11 |
| GATACG | 0.13 | **4.60x10^-2^** | 0.76 [0.57-0.99] | 0.20 |
| ACCGCA | 0.18 | 3.30x10^-1^ | 1.04 [0.78-1.39] | 0.18 |
| GATGCA | <0.01 | ***1.43x10^-5^*** | 0.30 [0.18-0.52] | 0.12 |
| ACCACA | <0.01 | 6.44x10^-2^ | 0.23 [0.05-1.09] | 0.03 |
| GATACA | 0.39 | ***9.36x10^-9^*** | 3.22 [2.17-4.76] | 0.20 |
| rs2981745-rs2981778-rs11523871-rs3013236-rs2981804-rs2277244-rs1052715 |  | ***5.63x10^-10^*** |  |  |
| CGATGTG | 0.02 | 9.62x10^-1^ | 0.98 [0.42-2.27] | 0.02 |
| CGATACG | 0.01 | ***3.23x10^-4^*** | 0.57 [0.42-0.77] | 0.23 |
| CGATGCG | 0.09 | 7.96x10^-2^ | 0.75 [0.54-1.03] | 0.10 |
| TACCGCA | 0.15 | 6.04x10^-1^ | 0.93 [0.70-1.23] | 0.20 |
| TACCACA | <0.01 | 7.63x10^-2^ | 0.32 [0.09-1.13] | 0.03 |
| TACCGCG | 0.11 | 7.88x10^-1^ | 0.95 [0.68-1.34] | 0.06 |
| CGATGCA | <0.01 | ***6.50x10^-6^*** | 0.16 [0.07-0.36] | 0.13 |
| CGATACA | 0.33 | **1.03x10^-3^** | 1.42 [1.15-1.75] | 0.17 |
| TGATACG | 0.02 | **5.94x10^-4^** | 22.00 [3.77-128.35] | <0.01 |

**Table S12. *DMBT1* gene markers in UC – Haplotype frequencies (HF), *P*-values, and odds ratios (OR) with 95% confidence intervals (CI).** *P-*values for individual haplotypes are presented for all haplotypes with a frequency of at least 1% in the whole sample and with an omnibus haplotype *P-*value < 0.05. Significant *P-*values (<0.05) are highlighted in **bold** and significant *P*-values robust to multiple testing (*P*<2.5x10^-3^ *for omnibus P*-values, *P*<4.8x10^-4^ for detailed haplotype *P-*values) are highlighted in ***bold italic***.
