# Supplementary material for: Intestinal DMBT1 Expression Is Modulated by Crohn’s Disease-Associated IL23R Variants and by a DMBT1 Variant Which Influences Binding of the Transcription Factors CREB1 and ATF-2
Source: PLoS One. 2013 Nov 5;8(11):e77773. doi: 10.1371/journal.pone.0077773 (PMC3818382; doi:10.1371/journal.pone.0077773)
Supplement: Table S13 — Analysis for epistasis between SNPs rs2981745, rs2981778, rs11523871 = p.Pro42Thr, rs3013236 = p.Leu54Ser, rs2981804, rs2277244 = p.His585Tyr, rs1052715 = p.Pro1707Pro within the DMBT1 gene and the SNP rs151181 in the IL27 gene region regarding CD/UC susceptibility. All P-values given are uncorrected for multiple comparisons. (DOC) [file pone.0077773.s017.doc]

| ***DMBT1* SNP** | ***IL27* (gene region) SNP rs151181:**  **Epistasis CD/UC** |
| --- | --- |
| rs2981745 | 0.8351/0.9995 |
| rs2981778 | 0.5009/0.9995 |
| rs11523871=p.Pro42Thr | 0.5182/0.9995 |
| rs3013236=p.Leu54Ser | 0.7073/0.9996 |
| rs2981804 | 0.1399/0.9998 |
| rs2277244=p.His585Tyr | 0.5023/0.9977 |
| rs1052715=p.Pro1707Pro | 0.3991/0.9911 |

**Table S13. Analysis for epistasis between SNPs rs2981745, rs2981778, rs11523871=p.Pro42Thr, rs3013236=p.Leu54Ser, rs2981804, rs2277244=p.His585Tyr, rs1052715=p.Pro1707Pro within the *DMBT1* gene and the SNP rs151181 in the *IL27* gene region regarding CD/UC susceptibility.** All *P*-values given are uncorrected for multiple comparisons.
