# Supplementary material for: Intestinal DMBT1 Expression Is Modulated by Crohn’s Disease-Associated IL23R Variants and by a DMBT1 Variant Which Influences Binding of the Transcription Factors CREB1 and ATF-2
Source: PLoS One. 2013 Nov 5;8(11):e77773. doi: 10.1371/journal.pone.0077773 (PMC3818382; doi:10.1371/journal.pone.0077773)
Supplement: Table S14 — Analysis for epistasis between SNPs rs2066844 = p.Arg702Trp, rs2066845 = p.Gly908Arg and rs2066847 = p.Leu1007fsX1008 in the NOD2 gene and the SNPs DMBT1 rs2981745 and rs2981804 within the DMBT1 gene regarding CD/UC susceptibility. All P-values given are uncorrected for multiple comparisons. (DOC) [file pone.0077773.s018.doc]

| ***NOD2* SNP** | ***DMBT1* rs2981745**  **Epistasis CD/UC** | ***DMBT1* rs2981804**  **Epistasis CD/UC** |
| --- | --- | --- |
| rs2066844=p.Arg702Trp | 0.9686/0.7862 | 0.1936/0.8109 |
| rs2066845=p.Gly908Arg | 0.0938/0.7779 | 0.0926/0.8890 |
| rs2066847=p.Leu1007fsX1008 | 0.5842/0.7442 | 0.7476/0.8634 |

**Table S14. Analysis for epistasis between SNPs rs2066844=p.Arg702Trp, rs2066845=p.Gly908Arg and rs2066847=p.Leu1007fsX1008 in the *NOD2* gene and the SNPs *DMBT1* rs2981745 and rs2981804 within the *DMBT1* gene regarding CD/UC susceptibility.** All *P*-values given are uncorrected for multiple comparisons.
