# Supplementary material for: Intestinal DMBT1 Expression Is Modulated by Crohn’s Disease-Associated IL23R Variants and by a DMBT1 Variant Which Influences Binding of the Transcription Factors CREB1 and ATF-2
Source: PLoS One. 2013 Nov 5;8(11):e77773. doi: 10.1371/journal.pone.0077773 (PMC3818382; doi:10.1371/journal.pone.0077773)
Supplement: Table S15 — Analysis for epistasis between SNPs rs1004819, rs7517847, rs10489629, rs2201841, rs11465804, rs11209026 = p.Arg381Gln, rs1343151, rs10889677, rs11209032, rs1495965 in the IL23R gene and the SNPs DMBT1 rs2981745 and rs2981804 within the DMBT1 gene regarding CD/UC susceptibility. All P-values given are uncorrected for multiple comparisons. Nominal significant P-values are indicated in bold. (DOC) [file pone.0077773.s019.doc]

| ***IL23R* SNP** | ***DMBT1* rs2981745**  **Epistasis CD/UC** | ***DMBT1* rs2981804**  **Epistasis CD/UC** |
| --- | --- | --- |
| rs1004819 | 0.2764/**0.0415** | 0.3253/**0.0313** |
| rs7517847 | 0.9675/0.9409 | 0.7367/0.6524 |
| rs10489629 | 0.1165/0.3355 | 0.6489/0.8983 |
| rs2201841 | 0.1846/0.4192 | 0.6615/0.7864 |
| rs11465804 | 0.7368/0.3675 | 0.7950/0.5429 |
| rs11209026=p.Arg381Gln | 0.9027/0.3790 | 0.8225/0.6761 |
| rs1343151 | 0.1977/0.5978 | 0.9198/0.7425 |
| rs10889677 | 0.1381/0.3627 | 0.5891/0.6413 |
| rs11209032 | 0.3867/0.8489 | 0.5732/0.3158 |
| rs1495965 | 0.9098/0.8099 | 0.3833/0.5429 |

**Table S15. Analysis for epistasis between SNPs rs1004819, rs7517847, rs10489629, rs2201841, rs11465804, rs11209026=p.Arg381Gln, rs1343151, rs10889677, rs11209032, rs1495965 in the *IL23R* gene and the SNPs *DMBT1* rs2981745 and rs2981804 within the *DMBT1* gene regarding CD/UC susceptibility.** All *P*-values given are uncorrected for multiple comparisons. Nominal significant *P-*values are indicated in bold.
