# Supplementary material for: Intestinal DMBT1 Expression Is Modulated by Crohn’s Disease-Associated IL23R Variants and by a DMBT1 Variant Which Influences Binding of the Transcription Factors CREB1 and ATF-2
Source: PLoS One. 2013 Nov 5;8(11):e77773. doi: 10.1371/journal.pone.0077773 (PMC3818382; doi:10.1371/journal.pone.0077773)
Supplement: Methods S1 — Supplementary methods. (DOC) [file pone.0077773.s020.doc]

**SUPPLEMENTARY METHODS**

**DNA extraction and genotyping of the *DMBT1* variants**

Seven *DMBT1* SNPs (rs2981745, rs2981778, rs11523871 [p.Pro42Thr], rs3013236 [p.Leu54Ser], rs2981804, rs2277244 [p.His585Tyr], rs1052715 [p.Pro1707Pro]) were genotyped by PCR and melting curve analysis using a pair of fluorescence resonance energy transfer (FRET) probes in a LightCycler® 480 instrument (Roche Diagnostics, Mannheim, Germany). The donor fluorescent molecule (fluorescein) at 3'-end of the sensor probe (or the anchor probe in the case of rs3013236 and rs2981804) is excited at its specific fluorescence excitation wavelength (533 nm) and the energy is transferred to the acceptor fluorescent molecule at the 5'-end (LightCycler Red 610, 640 or 670) of the anchor probe (or the sensor probe in the case of rs3013236 and rs2981804). The specific fluorescence signal emitted by the acceptor molecule is detected by the optical unit of the LightCycler 480 instrument. The sensor probe is exactly matching to one allele of each SNP, preferentially to the rarer allele, whereas in the case of the other allele there is a mismatch resulting in a lower melting temperature. The total volume of the PCR was 5 µl containing 25 ng of genomic DNA, 1 x Light Cycler 480 Genotyping Master (Roche Diagnostics), 2.5 pmol of each primer and 0.75 pmol of each FRET probe (TIB MOLBIOL, Berlin, Germany). Partly, two SNPs were analyzed in a multiplex reaction, the combinations were rs2981745 + rs11523871, rs3013236 + rs1052715 and rs2981778 + rs2981804. The PCR comprised an initial denaturation step (95°C for 10 min) and 45 cycles (50 cyles in the case of rs10065172) [95°C for 10°C sec, 60 °C (55 °C in the case of rs10065172) for 10 sec, 72°C for 15 sec]. The melting curve analysis comprised an initial denaturation step (95°C for 1 min), a step rapidly lowering the temperature to 40 °C and holding for 2 min, and a heating step slowly (1 acquisition/°C) increasing the temperature up to 95 °C and continuously measuring the fluorescence intensity. The results of the melting curve analysis have been confirmed by analyzing two patient samples for each possible genotype using sequence analysis. For sequencing, the total volume of the PCR was 100 µl containing 250 ng of genomic DNA, 1 x PCR buffer (Qiagen, Hilden, Germany), a final MgCl_2_ concentration of 2 mM, 0.5 mM of a dNTP mix (Sigma, Steinheim, Germany), 2.5 units of HotStar Plus Taq™ DNA polymerase (Qiagen) and 10 pmol of each primer (TIB MOLBIOL). The PCR comprised an initial denaturation step (95°C for 5 min), 35 cycles (denaturation at 94 °C for 30 sec, primer annealing at 60 °C for 30 sec, extension at 72 °C for 30 sec) and a final extension step (72 °C for 10 min). The PCR products were purified using the QIAquick PCR Purification Kit (Qiagen) and sequenced by a commercial sequencing company (Sequiserve, Vaterstetten, Germany). All sequences of primers and FRET probes used for genotyping and for sequence analysis are given in Tables S2 and S3.

## **Construction of *DMBT1* rs2981804 luciferase reporter plasmids**

All plasmids were constructed based on either the luciferase reporter plasmid pGL4.23 that contains a minimal promoter (minP) in front of the luciferase gene (low basal expression), or the pGL4.13 vector with a SV40 promoter (strong basal expression) (both from Promega, Mannheim, Germany) (summarized in supplementary Figures 1 and 2).

The genomic region surrounding rs2981804 was amplified from genomic DNA from HT-29 cells (rs2981804 genotype: AA) and DLD-1 cells (rs2981804 genotype: GG) with PCR primers (for sequences see table S14) introducing recognition sites for the restriction enzymes KpnI and XhoI (for cloning the fragment upstream, i.e. 5’ of the promoter into the multiple cloning site) or BamHI and XhoI restriction sites (for cloning the DMBT1 fragment 2 kb downstream, i.e. 3’ of the promoter). Resulting PCR products were double digested with KpnI/Xho or BamHI/XhoI, gel-purified and were ligated into gel-purified KpnI/XhoI or SalI/BamHI double-digested pGL4.23 (minP). For construction of plasmids containing both upstream (5’) and downstream (3’) DMBT1 inserts, a 5’-KpnI-3’-BamHI fragment of the upstream DMBT1 plasmid construct was ligated into a 5’-BamHi-3’-KpnI fragment of the plasmid containing the downstream DMBT1 fragment.

For the respective contructs in the SV40 vector background, DMBT1-fragment-containing minP plasmids were digested with BamHI and EcoRV and the respective fragment was ligated into the 5’-EcoRV-3’-BamHI digested SV40 backbone.

All PCR products and the final vector constructs were verified by sequencing confirming the presence of correct DMBT1 inserts differing in only one nucleotide (i.e. rs2981804 A or G).

**Bacterial transformation, colony PCR and plasmid isolation**

Competent E. coli bacteria (strain JM109) were prepared with the RbCl_2_ method. Briefly, a single colony was grown in 250 LB+20 mM MgSO_4_ until OD=0.4. Bacteria were harvested by centrifugation and were resuspended in 100 ml ice-cold TFB1 (30 mM potassium acetat, 100 mM RbCl, 10 mM CaCl_2_, 50 mM MnCl_2_, 15 % v/v glycerol, pH 5.8). After incubation on ice for 20 min, cells were pelleted by centrifugation and were resuspended in 10 ml ice-cold TFB2 (10 mM MOPS, 75 mM CaCl_2_, 10 mM RbCl, 15 % v/v glycerol, pH 6.5). After incubation on ice for 15 min, bacteria were aliquoted into pre-chilled cryovials and were snap-frozen in liquid nitrogen.

E, coli were transformed with 5 µl of a 10µl ligation reaction by heat-shock for 45 sec. Subsequently, they were plated on selective agar plates and grown overnight at 37°C. Colonies were screened by colony PCR for the respective inserts. Briefly, each colony was picked with a pipette tip, then first dipped into 10 µl of PCR mastermix containing all necessary PCR ingredients and the respective primers (table S14). Then the colony was streaked on a selective agar plate and incubated at 37°C. Hotstart PCR was performed and after identification of positive clones by PCR, the respective colonies were incubated in 4 ml of selective LB-Medium overnight and plasmid was isolated with the Miniprep Spin Kit (Qiagen). All isolated plasmids were verified by sequencing.

## **Transient transfection and luciferase assay**

DLD-1 and HT-29 (both from ATCC/LGC Standards, Wesel, Germany) cells were transiently transfected in 96 well plates (20,000 cells per well for DLD1, 15,000 cells per well for HT-29 cells) with 100 ng (DLD-1) or 200 ng (HT-29) plasmid using Lipofectamine LTX & Plus Reagent (Invitrogen) according to the manufacturer’s guidelines. Cells were co-transfected with a control Renilla luciferase vector (Promega) for normalization. Luciferase assay was performed 18-24 h post transfection with the Dual Glo Luciferase Assay System (Promega) and luminescence was detected in a TECAN Infinite M200 plate reader. Luciferase activity was normalized to Renilla luciferase activity in the respective cells.
